# Vaccination with a *Leishmania infantum HSP70-II* null mutant confers long-term protective immunity against *Leishmania major* infection in two mice models

**DOI:** 10.1371/journal.pntd.0005644

**Published:** 2017-05-30

**Authors:** José Carlos Solana, Laura Ramírez, Laura Corvo, Camila Indiani de Oliveira, Manoel Barral-Netto, José María Requena, Salvador Iborra, Manuel Soto

**Affiliations:** 1 Centro de Biología Molecular Severo Ochoa (CSIC-UAM), Departamento de Biología Molecular. Nicolás Cabrera 1, Universidad Autónoma de Madrid, Madrid, Spain; 2 Centro de Pesquisas Gonçalo Moniz (Fundação Oswaldo Cruz-FIOCRUZ). Salvador, Bahia, Brazil; University of Iowa, UNITED STATES

## Abstract

**Background:**

The immunization with genetically attenuated *Leishmania* cell lines has been associated to the induction of memory and effector T cell responses against *Leishmania* able to control subsequent challenges. A *Leishmania infantum* null mutant for the *HSP70-II* genes has been described, possessing a non-virulent phenotype.

**Methodology/Principal findings:**

The *L*. *infantum* attenuated parasites (*LiΔHSP70-II*) were inoculated in BALB/c (intravenously and subcutaneously) and C57BL/6 (subcutaneously) mice. An asymptomatic infection was generated and parasites diminished progressively to become undetectable in most of the analyzed organs. However, inoculation resulted in the long-term induction of parasite specific IFN-γ responses able to control the disease caused by a challenge of *L*. *major* infective promastigotes. BALB/c susceptible mice showed very low lesion development and a drastic decrease in parasite burdens in the lymph nodes draining the site of infection and internal organs. C57BL/6 mice did not show clinical manifestation of disease, correlated to the rapid migration of *Leishmania* specific IFN-γ producing T cells to the site of infection.

**Conclusion/Significance:**

Inoculation of the *LiΔHSP70-II* attenuated line activates mammalian immune system for inducing moderate pro-inflammatory responses. These responses are able to confer long-term protection in mice against the infection of *L*. *major* virulent parasites.

## Author summary

Despite numerous efforts made, a vaccine against leishmaniasis for humans is not available. Attempts based on parasite fractions or selected antigens failed to confer long lasting protection. On the other side, leishmanization, which consists in the inoculation of live virulent parasites in hidden parts of the body, is effective against cutaneous leishmaniasis in humans but objectionable in terms of biosafety. Some efforts have been made to design live vaccines to make leishmanization safer. A promising strategy is the development of genetically attenuated parasites, able to confer immunity without undesirable side effects. Here, we have employed an attenuated *L*. *infantum* line (*LiΔHSP70-II*) as a vaccine against heterologous challenge with *L*. *major* in two experimental models. Infection with *LiΔHSP70-II* parasites does not cause pathology and induces long-term protection based on the induction of IFN-γ producing T cells that are recruited rapidly and specifically to the site of challenge with the virulent parasites. These results support the idea of using attenuated parasites for vaccination.

## Introduction

Leishmaniases are a group of vector-borne diseases caused by the transmission of the protozoan parasite *Leishmania* in different mammalian hosts, during the blood meal of the invertebrate vectors (phlebotomine sandflies). Depending on the species of the parasite and the immune response of the host, the disease outcome varies from asymptomatic infections to clinical forms of the disease. The cutaneous forms of the disease (cutaneous leishmaniasis; CL) are characterized by the generation of disfiguring skin ulcers. In the Old World, is caused, among others, by the infection of *Leishmania major* species and together with the other forms of leishmaniasis is included in the list of neglected tropical diseases, affecting various developing countries [1]. Many efforts have been made in terms of prevention of leishmaniasis in the last decades, since it is believed that a vaccine against leishmaniasis is feasible given that patients recovered from the disease become resistant to new infections.

Mice infected with *L*. *major* have been widely used as experimental models for screening of vaccines. When BALB/c mice are experimentally challenged with *L*. *major* they suffer a progressive form of the disease, developing cutaneous lesions correlated to parasite multiplication at the site of infection as well as parasite dispersion to internal organs [2, 3]. Parasite specific IL-4 driven production of antibodies as well as the development of *Leishmania* related IL-10 deactivating responses are correlated with susceptibility [4]. On the other hand, C57BL/6 mice experimentally infected with *L*. *major* promote T-cell dependent IFN-γ production that results in the activation of infected macrophages to produce nitric oxide and to destroy the intracellular parasites [4]. Control of parasite mediated inflammation by regulatory T cells results in parasite persistent infection and resistance to reinfection [5]. Despite the fact that memory T cells can persist after parasite control [6], healed C57BL/6 mice lose their immunity to reinfection if they are manipulated to clear completely the parasites [7]. This has been taken as an evidence that persistence of live parasites is inevitably necessary for the maintenance of long-term protection [8]. In this context, the use of live attenuated parasites as vaccine candidates is a promising field of research. Live vaccines can induce adaptive immune responses relevant to protection by mimicking natural infection, without the adverse effects of leishmanization with virulent parasites.

Heat Shock Protein 70 (HSP70) plays a central role in both prokaryotic and eukaryotic cells because of its involvement in different aspects of protein metabolism (folding, assembly, activation, subcellular location, and so on) influencing many aspects of the cell biology, like cell growth and differentiation [9]. In *Leishmania*, the HSP70 also plays important roles in particular aspects affecting host-parasite interaction like virulence, drug resistance as well as in the induction of host immune responses (reviewed in [10]). There are two types of genes encoding HSP70 in *Leishmania infantum (= L*. *chagasi*), the causative agent of canine and human VL in the Mediterranean countries and in South America. Differences in their 3’ untranslated region (UTR) sequences have a great importance in the regulation of *Lihsp70* gene expression [11]. Since mRNAs having the 3’UTR-II are preferentially translated at 37°C [12], the expression of *Lihsp70-II* gene has been related to the response against the thermal stress caused by the parasite entry in the vertebrate host. Genetic elimination of the *Lihsp70-II* alleles resulted in a knock-out parasite line (*LiΔHSP70-II*) presenting a pleiotropic effect, influencing cell morphology, replication and, of special interest, virulence [13]. Hence, promastigotes (the form found in the insect vector) of the mutant line present some growth deficiencies in culture, and amastigotes show a limited capacity of multiplication inside macrophages, although *Lihsp70-II* gene deletion did not alter parasites uptake by these host cells. The inoculation of the mutant line did not produce any pathology in either hamster (highly susceptible for *L*. *infantum* infection) or in immune-deficient SCID mice, even though specific cellular responses were observed [13, 14]. In addition, the mutant *LiΔHSP70-II* was able to induce a short-term protection against *L*. *major* infection in BALB/c mice [14].

In this work, we have extended the study of these protective capacities analyzing short- and long-time protection after intravenous (i.v.) or subcutaneous (s.c.) infection with the *LiΔHSP70-II* line in the BALB/c-*L*. *major* model of progressive leishmaniasis. The immune correlates of protection have been also analyzed. The studies regarding the prophylactic properties of the *LiΔHSP70-II* line administration have been extended to the *L*. *major* infection resistant C57BL/6 mouse model. The vaccine-mediated robust protection shown in this line has been associated to the rapid recruitment of pre-existing CD4^+^ and CD8^+^ IFN-γ producing T cells to the site of *L*. *major* challenge.

## Results

### BALB/c mice infected with the *LiΔHSP70-II* line showed a persistent infection correlated to the induction of parasite specific IFN-γ predominant responses

**Fig 1:**
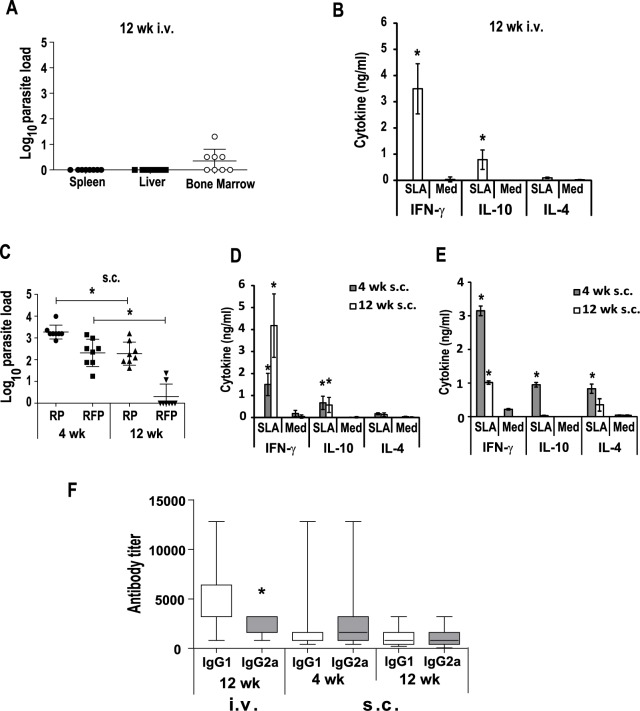
Evolution of parasite burdens and immune response induced after *LiΔHSP70-II* infection in BALB/c mice. Mice (n = 8 per group) were inoculated with PBS (Saline) or with 1 × 10^7^ *LiΔHSP70-II* promastigotes in the vein tail (i.v.) (A, B) or in the right footpad (s.c.) (C, D, E). In A and C, scatter plots of *LiΔHSP70-II* parasite burdens are shown including the mean ± standard deviation (SD). Parasite loads were analyzed by limiting dilution at week 12 (12 wk) for i.v. inoculated animals (A) and at week 4 (4 wk) and 12 wk for s.c. inoculated ones (C) in the spleen (parasites per total organ), liver (parasites per g) or bone marrow (parasite per 1 × 10^7^ cells) (A). In (C) parasite burdens in the right popliteal lymph node (RP, parasites per total lymph node) and in the right footpad (RFP, parasites per total footpad) are shown. * (*P* < 0.05) indicates the statistical differences in 4 wk and 12 wk parasite burdens determined by a Mann-Whitney test. Spleen cell cultures for i.v. (B) or s.c. (D), or right popliteal lymph node cell cultures for s.c. (E) vaccinated animals were established at the indicated times after the inoculation of the *LiΔHSP70-II* line. The presence of cytokines in supernatants was measured after growing cells in the absence (Med) or in the presence of *L*. *infantum* SLA. In B and D, data show the mean ± SD of at least 8 mice per group. In E, data show the mean ± standard error of mean (SEM) of two independent assays performed with pooled cells from 8 mice. * *P* < 0 .05 shows statistical differences between SLA-stimulated and non-stimulated cells (unpaired Student t-test). The IgG1 and IgG2a reciprocal end-point titers against *L*. *infantum* SLA were analyzed by ELISA at the indicated times for i.v and s.c. inoculated mice, and represented as whisker (min to max) plots (F). * (*P* < 0.05) indicates the statistical differences between IgG1 and IgG2a anti-SLA titers (Kruskal-Wallis test and Dunn's Multiple Comparison post-test). No parasite loads or SLA-specific antibodies or cytokines were detected in mice receiving saline. Results are representative of at least two independent experiments.

In a previous work, it was described that i.v. infection with the *LiΔHSP70-II* attenuated parasites (vaccination) resulted in short-term protection against *L*. *major* infection when challenged four weeks after vaccination [14]. This protection was correlated to the presence of the attenuated parasites in the liver and spleen of the protected animals [14]. Here, we firstly analyzed the evolution of the attenuated parasites in the internal organs of the i.v. vaccinated mice for a longer period of time. Parasites detected at week 4 after vaccination (S1 Fig panel A) [14] were undetectable in the spleen or liver at week 12 after vaccination (Fig 1A). However, in the bone marrow (BM), although at week 12 post vaccination parasite burden significantly decreased compared to the 4^th^ week (P < 0.05; unpaired T-test), we still detected parasites in 4 out of 8 mice (Fig 1A). Vaccination induced a *Leishmania*-specific cellular response that was revealed after stimulation with soluble leishmanial antigen (SLA) of spleen cells. We detected SLA-specific secretion of IFN-γ and IL-10 to a lesser extent, both at the 4^th^ (S1 Fig panel B) or at the 12^th^ (Fig 1B) week post-vaccination. Alternatively, we administered s.c. the *LiΔHSP70-II* based vaccine to analyze its immunogenicity in a different vaccination setting. No parasites were observed in liver, spleen and BM of these animals at any time. Parasites were detected in the right popliteal which corresponds to the draining lymph node (DLN) close to the inoculation site (Fig 1C). Although parasite numbers decreased over time (*P* = 0.003) (Fig 1C), all animals presented live parasites in the DLN at week 12. The analysis of the parasite burden in the site of vaccination (right footpad) revealed the presence of live parasites in all the animals at week 4 after vaccination, that decreased significantly at week 12 (*P* = 0.0013). At this time, parasites were only detected in two out of eight vaccinated animals (Fig 1C). Interestingly, although only a local infection occurred, the immune response detected in the spleen was similar in profile and magnitude to that found in the i.v. vaccinated animals, with a predominant SLA-specific production of IFN-γ, which was more prominent at 12 weeks after vaccination (Fig 1D). The analysis of the parasite-specific production of cytokines by cells derived from the DLN (right popliteal) showed also an IFN-γ predominant response, higher in magnitude at the short-term (Fig 1E), coinciding with the presence of high numbers of the attenuated parasites (Fig 1C). In addition, at week 4 after vaccination, IL-10 and IL-4 were detected in SLA-stimulated cultures (Fig 1E). Regarding the humoral response elicited by vaccination, i.v. inoculated mice showed a mixed IgG response at week 4 (S1 Fig panel C and [14]) and at week 12 after vaccination (Fig 1F), with titers that decreased over time and were predominantly of the IgG1 isotype rather than IgG2a. Very low levels of anti-SLA IgG1 and IgG2a levels were detected in the sera of s.c. vaccinated animals, especially at long-term (Fig 1F).

**Fig 2:**
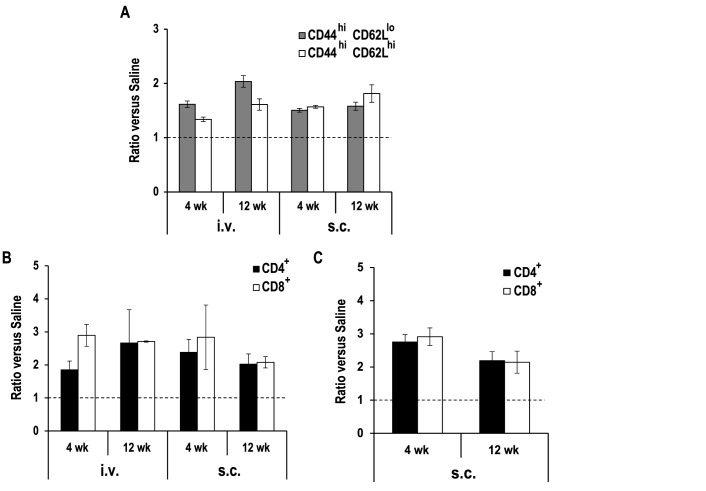
Analysis of splenic T cell populations in vaccinated mice. Mice (n = 4 per group) were inoculated with PBS (Saline; s.c.) or with 1 × 10^7^ *LiΔHSP70-II* promastigotes in the vein tail (i.v.) or in the right footpad (s.c.). After 4 weeks (vaccinated) and 12 weeks (saline and vaccinated), T cells from the spleen were studied in pool by flow cytometry. Animals were age matched at the moment of the analysis. In (A) analyzed CD4^+^ T cells were: antigen-experienced cells CD44^high^, central memory T cells (Tcm) CD44^high^CD62L^high^ and effector T cells or effector memory T cells (Teff/Tem) CD44^high^CD62L^low^. Spleen cells (B) or right popliteal lymph node cells (C) of the indicated groups were ex vivo stimulated by anti-CD3/anti-CD28 and treated with brefeldin A to block cytokine secretion. Cells were characterized by using anti-CD4 and anti-CD8 antibodies as well as by intracellular staining for IFN-γ. The ratio between the percentages of CD4^+^ or CD8^+^ producing IFN-γ cells in vaccinated animals versus saline ones, derived from two independent experiments is shown (mean ± standard error of mean).

Further, we explored the percentages of T cell populations in the spleen from control and vaccinated animals by flow cytometry. Two subsets of helper T cells (CD3^+^CD4^+^) were characterized according to the presence of CD44 and CD62L molecules. All vaccinated groups showed an increase in the percentage of antigen-experienced CD4^+^ cells (CD44^high^) compared with the control (Saline) (Fig 2A). Comparison between inoculation routes indicates that similar levels of CD4^+^ central memory T cells (Tcm; CD44^high^CD62L^high^) were found in both groups. On the other hand, i.v. immunization elicited further expansion of CD4^+^ effector memory (Tem) or effector (Teff) T cells (CD44^high^CD62L^low^) compared with s.c. route (Fig 2A; S2 Fig panel A). Also, we determined that i.v. and s.c. vaccinated mice exhibited a higher frequency of both CD4^+^ and CD8^+^ IFN-γ producing splenic T cells compared to the unvaccinated group after in vitro stimulation with anti-CD3/anti-CD28 antibodies (Fig 2B; S2 Fig panel C). This increment was also observed in the DLN (right popliteal) of the s.c. group (Fig 2C; S2 Fig panel D).

Altogether, these data allowed concluding that inoculation of the *LiΔHSP70-II* attenuated line, independently of the inoculation route, caused a persistent regressive infection that resulted in the induction of Tcm and Tem/Teff cell responses. Vaccinated animals showed a parasite dependent production of IFN-γ in which CD4^+^ and CD8^+^ T cells seem to be involved.

### The *LiΔHSP70-II* based vaccine is able to induce short- and long-term protection against a *L*. *major* infective challenge in BALB/c mice

**Fig 3:**
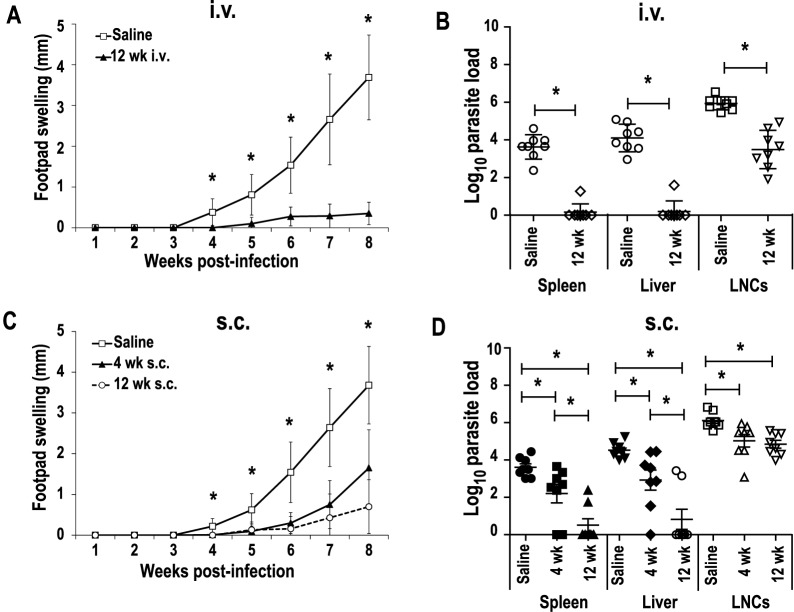
Mice vaccinated with the attenuated *LiΔHSP70-II* line showed protection against an infective challenge with *L*. *major*. BALB/c mice (n = 8 per group) inoculated with PBS (Saline) or with 1 × 10^7^ *LiΔHSP70-II* promastigotes in the vein tail (i.v.) (A, B) or in the right footpad (s.c.) (C, D) were challenged with 5 × 10^4^ stationary-phase *L*. *major* promastigotes in the left footpad at week 4 (s.c.) or at week 12 after vaccination (i.v. and s.c.). Footpad swelling was monitored weekly. Mean ± standard deviation (SD) is shown (A, C). *L*. *major* parasite burdens were determined by limiting dilution in the spleen, liver and in the draining lymph node (left popliteal). Scatter plots with the individual number of parasite per total organ (spleen or lymph nodes) or per g of liver are shown including the mean ± SD (B, D). * (P < 0 .05) shows the statistical differences determined by the one-way ANOVA test followed by the Tukey post-test. Results are representative of two independent experiments.

To analyze the effect of the live vaccine on the development of a progressive leishmaniasis, BALB/c mice vaccinated with the *LiΔHSP70-II* line administered i.v. were challenged with *L*. *major* parasites (5 × 10^4^ stationary phase promastigotes) s.c. in the left footpad. As a control, mice inoculated with PBS at the time of the vaccination were also infected with *L*. *major*. Infective challenge was performed short- (4 weeks) or long-term (12 weeks) after vaccination. Fig 3A shows that i.v. vaccination also induced long-term protection, since very low footpad swelling was observed in the vaccinated groups, as it was reported for the short-term [14] and confirmed in this work (S1 Fig panel D). The lack of lesions correlated to a decrease in *L*. *major* parasite burdens relative to saline controls in all analyzed organs (Fig 3B). Regarding the presence of the parasite in visceral organs, a significant decrease was observed in both vaccinated groups with respect unvaccinated controls (*P <* 0.05). In the 4 weeks group, 50% (4/8) and 37.5% (3/8) of the mice had undetectable parasites in the spleen or liver, respectively (S1 Fig panel E). In the 12 weeks group the percentage of negative mice reached values of 87.5% (7/8) in both organs (Fig 3B). Data comparison among control and vaccinated mice groups revealed a higher decrease in parasite loads at the DLN for long-term infected mice (12 weeks; 2.4-log reduction; *P <* 0.001) (Fig 3B) than in the short-term group (4 weeks 1.2-log reduction; *P <* 0.01) (S1 Fig panel E). A decrease in the evolution of footpad swelling was also observed in mice s.c. vaccinated compared to control mice (saline group) (Fig 3C). Although no significant differences were found in the cutaneous lesions developed between mice of the 4 weeks and 12 weeks groups, short-term infected mice showed a progressive evolution of the footpad swelling evident from week 7 to week 8. At that time, mice from all groups were euthanized because of the appearance of necrotic lesions in some mice of the control group. The clinical lesions evolution can be taken as an indication of a partial short-term protection driven by the s.c. inoculation of the *LiΔHSP70-II* line that was improved long-term. Determination of *L*. *major* parasite burdens in the spleen and liver (Fig 3D) also demonstrated a significant decrease compared to control animals in both s.c. vaccinated groups (*P* < 0.05 and *P* < 0.001, for 4 weeks and 12 weeks, respectively). In this case, most of the mice of the 4 weeks group were positive for *L*. *major* parasites in the spleen (75%, 6/8) or liver (87.5%, 7/8), supporting the partial protection concluded from the clinical data. On the other hand, only two mice from the long-term protected groups were positive for live parasites in both internal organs (Fig 3D). Regarding parasite loads in the left popliteal DLNs, a significant decrease (*P* < 0.05 and *P* < 0.01, for 4 weeks and 12 weeks respectively) was obtained when vaccinated mice were compared to control mice. Similar decreased values with respect saline controls were found in the parasite numbers for both vaccinated groups in the liver and in the spleen (1-Log and 1.3-Log for 4 weeks and 12 weeks groups, respectively) (Fig 3D).

On the other hand, we evaluated the presence of the *LiΔHSP70-II* attenuated parasites in different organs and tissues of the vaccinated mice after *L*. *major* challenge (S3 Fig panel A). These analyses indicated that challenge with infective parasites did not reactivate the infection of the attenuated line. For i.v. vaccinated mice (S3 Fig panel B) no *LiΔHSP70-II* parasites were found in the internal organs, except parasites detected in the BM at short-term that were equivalent to those determined in the 12 wk vaccinated group (Fig 1A). Interestingly, BM became negative for *LiΔHSP70-II* parasites at week 20 (S3 Fig panel B). In addition, no attenuated parasites were found in the left popliteal LNs, in spite of the presence of *L*. *major*. Regarding the s.c. vaccinated mice, we only observed the persistent presence of the *LiΔHSP70-II* parasites in the LN draining the site of vaccination at week 20 (S3 Fig panel C).

**Fig 4:**
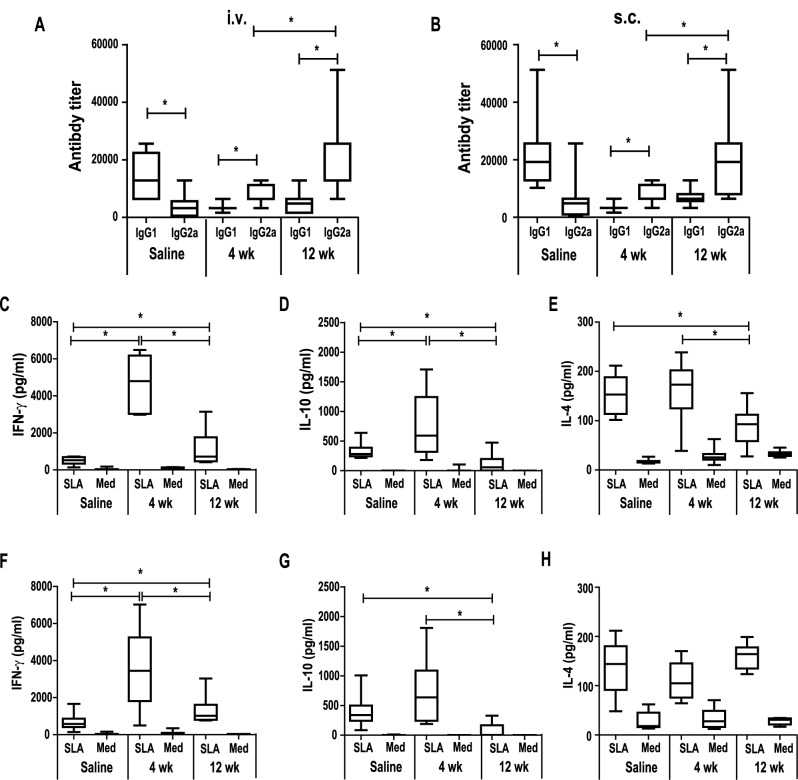
Humoral and cellular parasite induced responses after *L*. *major* challenge. BALB/c mice (n = 8 per group) inoculated with PBS (Saline) or with 1 × 10^7^ *LiΔHSP70-II* promastigotes in the vein tail (i.v.) (A, C, D, E) or in the right footpad (s.c.) (B, F, G, H) were infected with 5 × 10^4^ stationary-phase *L*. *major* promastigotes in the left footpad at week 4 or at week 12 after vaccination. Animals were euthanized 8 weeks after *L*. *major* challenge. IgG1 and IgG2a reciprocal end-point titers against *L*. *major* SLA were determined by ELISA (A, B). Results are shown as whisker (min to max) plots of at least 8 mice per group. * (P < 0.05) indicates the statistical differences between IgG1 and IgG2a anti-SLA titers within each group or differences in IgG2a between short- and long-term protected mice (Mann-Whitney test). For cytokine determinations spleen cells were cultured in the absence (Med) or in the presence of *L*. *major* SLA. Levels of IFN-γ (C, F), IL-10 (D, G) and IL-4 (E, H) were assessed by ELISA in culture supernatants and shown as whisker (min to max) plots of at least 8 mice per group. * (P < 0.01) shows the statistical differences of saline and short-term or long-term vaccinated mice groups, or the statistical differences between short-term and long-term vaccinated mice groups (Mann-Whitney test). Results are representative of two independent experiments.

Once we determined that vaccination with *LiΔHSP70-II* parasites induced protection for both, clinical manifestations and parasitemia, we analyzed the immune correlates of protection. For that, we next determined humoral and cellular responses specific for the parasite using SLA in ELISA assays and for cell stimulation in all vaccinated groups and their corresponding saline controls, 8 weeks after *L*. *major* challenge. Protection correlated with an IgG subclass redirection to Th1-related IgG2a subclass of SLA-specific antibodies in the vaccinated mice that were mainly of the IgG1 subclass in saline controls (Fig 4A and 4B, for i.v. and s.c., respectively). The magnitude of the IgG2a response was higher in long-term protected mice than in short-term groups (*P* < 0.0018 and *P* < 0.023 for i.v. and s.c., respectively). Cellular responses against SLA in the *L*. *major* infected mice were determined by stimulating spleen cells from mice receiving saline or the attenuated line (4 weeks and 12 weeks groups) i.v. (Fig 4C–4E) or s.c. (Fig 4F–4H). In agreement with the Th1-like profile of the humoral response, a SLA-dependent IFN-γ predominant response was found in all protected groups reaching higher *P* values with respect to saline control in short-term protected mice (*P* = 0.0003 and *P* = 0.001 for i.v and s.c. groups, respectively) than long-term groups (*P* = 0.026 and *P* = 0.021 for i.v and s.c. groups, respectively). Interestingly, long-term protected mice showed a concomitant significant decrease in the IL-10 levels secreted after stimulation with parasite proteins when compared to saline controls (*P* = 0.0006 and *P* = 0.0063 for i.v and s.c. groups, respectively) (Fig 4D and 4G). This decrease was absent in short-term protected mice. On the contrary, short-term protected group secreted higher amounts of IL-10 than control mice although only significant differences were observed in the i.v. vaccinated group (*P* = 0.0361) (Fig 4D). When IL-4 production was analyzed (Fig 4E and 4H), a decrease in the levels of SLA-specific IL-4 in the culture supernatant was only found when saline controls were compared to long-term i.v. vaccinated mice (*P* = 0.003) (Fig 4E). The cytokine production specific for SLA was higher in short- than in long-term protected mice: *P* = 0.0003, *P* = 0.0002 and *P* = 0.0062 for IFN-γ, IL-10 and IL-4, respectively in the i.v. group (Fig 4C, 4D and 4E) and *P* = 0.022 and *P* = 0.0072 for IFN-γ and IL-10, respectively in the s.c. group (Fig 4F and 4G).

### C57BL/6 mice s.c. vaccinated with *LiΔHSP70-II* attenuated parasites showed no clinical manifestations when challenged with *L*. *major*

**Fig 5:**
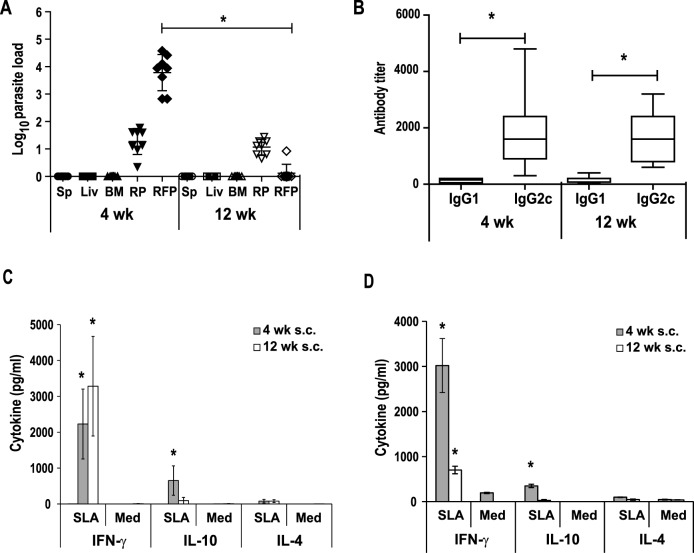
C57BL/6 mice chronically infected with the *LiΔHSP70-II* showed a Th1 like response against *Leishmania*. C57BL/6 mice (n = 8) were infected with 1 × 10^7^ *LiΔHSP70-II* promastigotes in the right footpad (s.c.). *LiΔHSP70-II* parasite burdens were analyzed at week 4 or at week 12 after vaccination by limiting dilution in the spleen (Sp, parasites per total organ), liver (Liv; parasites per g), bone marrow (BM; parasites per 1 × 10^7^ cells), in the right popliteal lymph node (RP; parasites per total lymph node) or right footpad (RFP; parasites per total footpad) (A). Scatter plots are shown including the mean ± standard deviation (SD). No statistical differences were found in the parasite burdens in the RP from week 4 to week 12 whereas a significant decrease was shown in the RFP (unpaired Student t-test). At week 4 or at week 12 after vaccination, animals were euthanized and spleen cell cultures and sera were prepared. The IgG1 and IgG2c reciprocal end-point titer against *L*. *infantum* SLA was determined by ELISA and represented as whisker (min to max) plots (B). * (P < 0.05) indicates the statistical differences between IgG1 and IgG2c anti-SLA titers within each group (Mann-Whitney test). For cytokine determinations spleen cells (C) or RP cells (D) were cultured in the absence (Med) or in the presence *L*. *infantum* SLA. Levels of IFN-γ, IL-10 and IL-4 were assessed by ELISA in culture supernatants. Mean ± standard deviations (SD) are shown in C and mean ± standard error of mean (SEM) are shown in D. * (P < 0.01) shows the statistical differences between SLA-stimulated and non-stimulated cells (unpaired Student t-test). No parasite loads or SLA-dependent cytokines were detected in saline immunized mice. Results are representative of two independent experiments.

Since s.c. inoculation of the attenuated parasites was able to long-term protect BALB/c mice against a *L*. *major* infective challenge, we decided to analyze the prophylactic properties of the s.c. administered vaccine in C57BL/6 mice. Inoculation of 1 × 10^7^ *LiΔHSP70-II* promastigotes in the right footpad of mice produced a chronic infection in the DLN (right popliteal) as revealed by the analysis of parasite burdens at week 4 and week 12 post-vaccination, whereas parasites were found in the site of vaccination (right footpad) at week 4 after vaccination but disappeared at week 12 (in 87.5% of the mice; 7/8 mice) (Fig 5A). Attenuated parasites were absent of the internal organs (Fig 5A). The presence of a persistent number of *LiΔHSP70-II* parasites in the popliteal lymph node draining the site of the attenuated line inoculation was maintained after *L*. *major* challenge up to 25 weeks (S4 Fig). Short-term and long-term vaccinated mice showed an IgG2c predominant antibody response against the parasite (Fig 5B) and their spleen cells secreted IFN-γ after in vitro stimulation with *L*. *infantum* SLA (Fig 5C). Contrary to the long-term vaccinated group, short-term vaccinated mice secreted detectable levels of IL-10 in response to SLA (Fig 5C). A SLA-dependent production of IFN-γ was detected in the popliteal LN culture supernatants from both vaccinated groups, higher in magnitude at week 4 after infection along with IL-10 production (Fig 5D).

**Fig 6:**
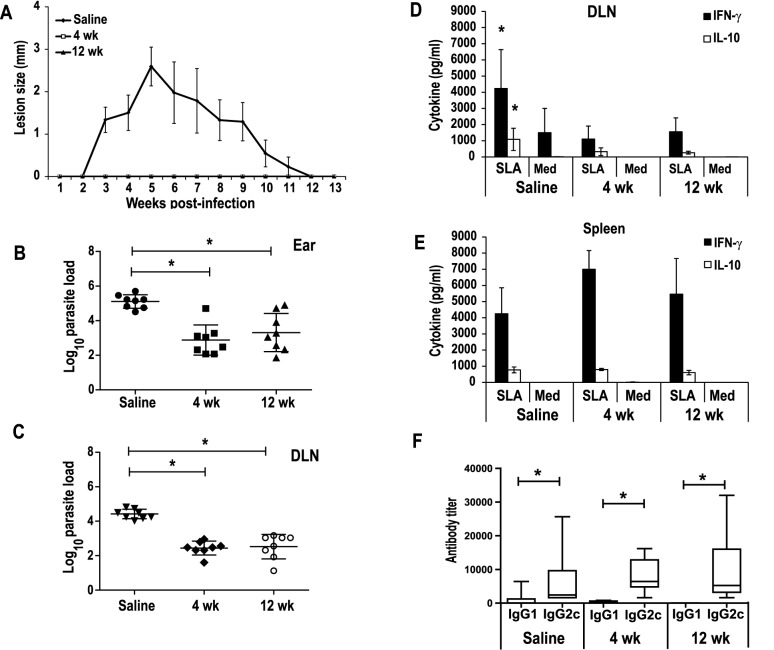
Evolution of *L*. *major* infection in C57BL/6 vaccinated mice. Animals (n = 8 per group) inoculated with PBS (Saline) or with 1 × 10^7^ *LiΔHSP70-II* promastigotes in the right footpad (s.c.) were challenged with 1 × 10^3^ metacyclic *L*. *major* promastigotes in the dermis of both ears at week 4 or at week 12 after vaccination. (A) Ear lesion diameter was monitored weekly (16 ears from week 1 to 5 and 8 ears from week 6 to 13). Mean ± standard deviation (SD) is shown. *L*. *major* parasite burdens were determined at week 5 after *L*. *major* challenge by limiting dilution in the ears (n = 8 per group) (B) or in the retromandibular draining lymph node (n = 8 per group) (C). Scatter plots with the individual number of parasite per total organ are shown including the mean ± standard deviation (SD), * (P < 0.05) shows the statistical differences determined by the one-way ANOVA test followed by the Tukey post-test. For cytokine determinations the DLNs (D) or the spleen (E) from mice sacrificed at week 5 after *L*. *major* challenge (n = 4 mice) cells were cultured in the absence (Med) or in the presence *L*. *major* SLA. Levels of IFN-γ and IL-10 were assessed by ELISA in culture supernatants. The means ± SD are shown. * (P < 0.01) shows the statistical differences among saline and short-term or long-term vaccinated mice groups (unpaired Student t-test). The IgG1 and IgG2c reciprocal end-point titer against *L*. *major* SLA was determined by ELISA at week 5 after *L*. *major* challenge (n = 8 mice per group) and represented as whisker (min to max) plots (F). * (P < 0.05) indicates the statistical differences between IgG1 and IgG2c anti-SLA titers within each group (Mann-Whitney test). Results are representative of at least two independent experiments.

Most importantly, neither short-term nor long-term vaccinated mice showed any inflammatory lesion when challenged with 1 × 10^3^ *L*. *major* metacyclic promastigotes in the ear dermis (Fig 6A). At week five after challenge, *L*. *major* burdens were similar in short- and long-term vaccinated mice, showing a 1.5-Log (Fig 6B) and 2-Log (Fig 6C) reduction in the ears and DLNs, respectively, when compared to the saline controls. No *L*. *major* parasites were found in visceral organs (liver or spleen). Retromandibular LNs cells from mice of the saline group were able to secrete higher amounts of cytokines than vaccinated mice when analyzed at week 5 after *L*. *major* challenge (Fig 6D). A significant increment in IFN-γ (*P* = 0.023 relative to week 4 and *P* = 0.041 relative to week 12) and IL-10 (*P* = 0.022 relative to week 4 and *P* = 0.012 relative to week 12) was observed in LN culture supernatants from cells obtained from saline controls when compared with both vaccinated samples after in vitro stimulation with SLA. As it was expected because of the presence of inflammatory lesions, control mice DLN cells secreted IFN-γ in the absence of SLA stimulation whereas this cytokine was absent in unstimulated cultures stablished from vaccinated mice (Fig 6D). On the other hand, similar amounts of IFN-γ were observed among the three groups when the stimulation assay was performed in spleen cell cultures (Fig 6E). These data, besides the presence of IgG2c anti-SLA antibodies in all groups (Fig 6F) allowed the conclusion that all mice groups have a systemic Th1 response against the parasite. In controls, the lymph node inflammatory response was related to the presence of high numbers of *L*. *major* parasites (i.e. showing inflammatory lesions). In the vaccinated mice the limited infection in the DLNs was correlated to a lower IFN-γ local response. Thus, systemic response mounted by the asymptomatic infection of the attenuated line, resulted in a protective response against *L*. *major* challenge in the absence of pathological lesions.

### C57BL/6 vaccinated mice mounted a moderate and rapid IFN-γ response able to control *L*. *major* intradermal infection progression

**Fig 7:**
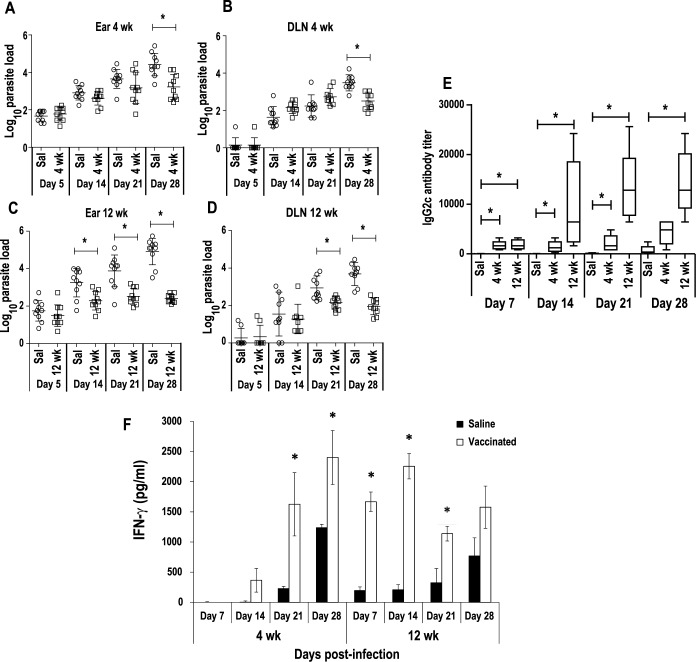
Vaccinated mice mounted an early immune response against *Leishmania*. Animals inoculated with PBS (Saline) or with 1 × 10^7^ *LiΔHSP70-II* promastigotes in the right footpad (s.c.) were challenged with 1 × 10^3^ metacyclic *L*. *major* promastigotes in the dermis of both ears at week 4 and at week 12 after vaccination. Mice (n = 5) per group were weekly sacrificed to analyze parasite loads in both ears (A and C), or in the corresponding retromandibular draining lymph nodes (B and D). Scatter plots with the individual number of parasites per total organ are shown including the mean ± standard deviation (SD). * (P < 0 .05) shows the statistical differences determined by the unpaired Student t-test. The IgG2c reciprocal end-point titer against *L*. *major* SLA was analyzed by ELISA weekly after *L*. *major* challenge and represented as whisker (min to max) plots (E). * (P < 0.05) indicates the statistical differences in IgG2c anti-SLA titers among the three groups (Kruskal-Wallis test and Dunn's Multiple Comparison post-test). Level of *L*. *major* dependent IFN-γ produced in retromandibular cell cultures (F). Mean ± SD are shown. * (P < 0.01) shows the statistical differences between saline vaccinated mice groups (unpaired Student t-test).

Next, we tested whether the Th1 response induced by the inoculation of the attenuated line is able to anticipate the response against *L*. *major* parasites in the site of infection, resulting in a non-pathological protection. For that purpose, C57BL/6 mice were inoculated with the attenuated line 4 weeks or 12 weeks before, and then challenged in the ears with 1 × 10^3^ metacyclic forms of *L*. *major*. A progressive increment in the number of parasites found in the ear an in the DLNs was observed in the saline groups up to day 28 post-challenge (Fig 7A–7D). On the contrary, the number of parasites were stabilized in vaccinated mice 28 days after challenge in the short-term protected mice (Ear Fig 7A; DLNs Fig 7B), and from day 14 in the ear or day 21 in the DLNs in the long-term group (Fig 7C and 7D, respectively). Parasite replication control was correlated to the early presence of circulating anti-SLA IgG2c antibodies in the sera from vaccinated mice after *L*. *major* challenge (Fig 7E). A higher reactivity that was not statistically different was observed in mice of the long-term group when compared to the short-term vaccinated animals. In addition, short-term and especially long-term vaccinated mice were able to mount earlier cellular responses against the parasite than control mice, as demonstrated by the levels of IFN-γ secreted to the culture supernatants in the three groups, after in vitro stimulation with SLA of the cells obtained from *L*. *major* infected DLNs (Fig 7F).

**Fig 8:**
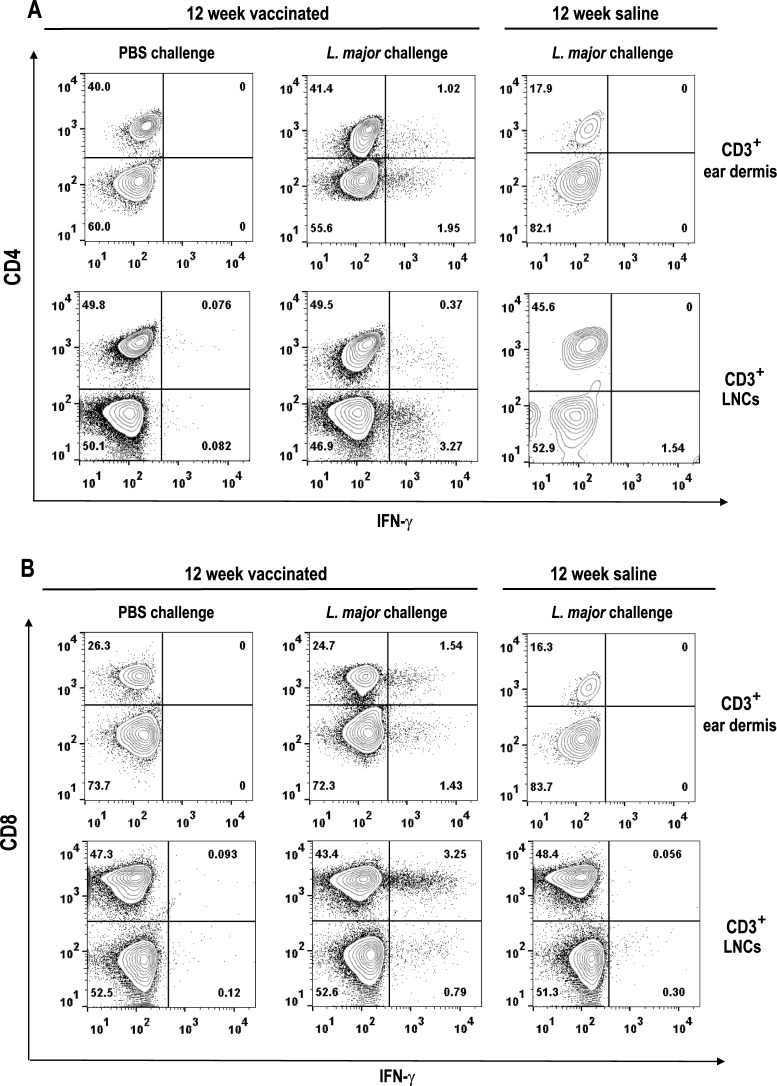
Analysis of the early response after *L*. *major* challenge in the site of infection. C57BL/6 mice were inoculated with PBS (12 week saline group; n = 6) or with 1 × 10^7^ *LiΔHSP70-II* promastigotes (12 week vaccinated group; n = 10) in the right footpad (s.c.). After 12 week, vaccinated animals were inoculated with PBS (PBS challenge; n = 5) or infected with 1 × 10^5^ *L*. *major* metacyclic promastigotes (*L major* challenge; n = 5) in the dermis of both ears. Mice inoculated in the right footpad (s.c.) with PBS (12 week saline group) were also challenged with *L*. *major* in the ear dermis. Three days after infection T cells from the ears and retromandibular lymph nodes were analyzed in pool by flow cytometry after anti-CD3/anti-CD28 ex vivo stimulation. Contour plots of IFN-γ intracellular staining for CD4^+^ (A) or CD8^+^ (B) cells (gated on CD3^+^) are shown. Numbers represent the percentage of each population.

Finally, we analyzed IFN-γ synthesis at the site of *L*. *major* challenge upon long-term vaccination (Fig 8 and S5 Fig). For that purpose, mice inoculated with the attenuated parasites and their corresponding saline controls were challenged, 12 weeks after vaccination in the ear dermis with *L*. *major* (1 × 10^5^). As an additional control, a group of vaccinated mice was i.d. injected with PBS in the ears at the time of *L*. *major* challenge. Three days after inoculation, the presence of IFN-γ secreting cells in the ears and the DLNs was analyzed by flow cytometry. Both CD4^+^ (Fig 8A) and CD8^+^ (Fig 8B) IFN-γ secreting T cells were detected in mice vaccinated with the attenuated line shortly after *L*. *major* challenge. Such cells were absent from the site of infection of unchallenged vaccinated mice, or in non-vaccinated and infected animals.

## Discussion

The fact that patients recovered from CL disease are usually resistant to reinfection has been taken as an indication that a vaccine against this form of the disease is feasible. Historically, leishmanization (inoculation of live virulent *L*. *major* parasites) was employed to induce immunity against CL, and although it is currently in disuse, the practice is coming back in regions of high incidence because of its effectiveness [15–17]. The use of murine models of CL demonstrated that leishmanization protects C57BL/6 resistant mice from vector transmitted *L*. *major* infection contrary to vaccines based on parasite extracts [18] which failed to protect against natural infection. Also, some limitations were observed in the prophylactic properties of the most evolved recombinant molecules based vaccines showing different protection degree in distinct murine models of CL due to sand fly transmitted infection [19, 20]. In addition, to maintain immunity, protein-based vaccines require boosting doses, since transient effector T cell responses preclude the induction of long-term immunity [21]. On the contrary, the balanced effector/memory T response induced by the infection with virulent parasites can be maintained by parasite persistence, resulting in long-term immunity [16, 17]. A possible limitation of leishmanization has emerged after analyzing the influence of *L*. *major* challenge on the evolution of leishmaniasis caused by other species of *Leishmania* in murine models. Whereas cutaneous infection with *L*. *major* provided heterologous protection against VL due to *L*. *infantum* infection in C57BL/6 mice [22] the IL-4 mediated humoral response elicited against the parasite by leishmanization in BALB/c mice caused an aggravation of VL disease when ‘leishmanized’ mice were challenged with *L*. *infantum* [23].

In recent years, vaccination with genetically attenuated parasites is being contemplated as a promising alternative to leishmanization, avoiding the problems derived from using non-attenuated parasites [17, 24]. As reviewed in [25] genetically modified *L*. *major* attenuated lines have shown some limitations when tested as vaccines against CL. Thus, protection against *L*. *major* challenge induced in resistant or susceptible mice by the inoculation of the *L*. *major* conditional auxotroph due to targeted deletion of the dihydrofolate reductase-thymidylate synthase gene (*Lmdhfr-ts*^*-/-*^) [26] was not reproduced in a primate model [27]. In addition, vaccines based on the *L*. *major* line lacking phophoglycans (*LmLpg2*^*-*^) presented differences in the induced protective immunity depending on the murine model assayed [28, 29]. Also, infection of a *L*. *major* genetically modified arginase deficient line resulted in a chronic disease in which lesions did not disappear in the resistant mouse strain [30]. The most efficient genetically modified vaccine for CL was constructed in *L*. *major* by the inclusion of two suicide genes (*Lmtkcd*^*+/+*^) that render the parasite susceptible to ganciclovir and 5-flurocytosine [31]. This vaccine has been tested to be effective in the BALB/c [31] or in the C57BL/6 models [32], but treatment needs to be administered to recipients after vaccination complicating the vaccine schedule. As an alternative, in this work we propose the use of single dose of a live vaccine based on a *L*. *infantum* genetically attenuated line [13] to induce protection against CL.

Regarding the evolution of the attenuated parasite burdens in the vertebrate host, inoculation of *LiΔHSP70-II* in BALB/c mice using the i.v. route led to a systemic infection with a pattern of parasite clearance with time post-infection in all internal organs. Importantly, challenge with infective *L*. *major*, did not produce the reactivation of the attenuated line. Similarly, i.v. inoculation of the latest and more promising attenuated vaccines based on *L*. *donovani* (*LdCen*^-/-^) deficient in centrin, a calcium binding cytoskeletal protein [33] or *Ldp27*^*-/-*^; lacking a protein forming part of the active cytochrome c oxidase complex [34]) produced a transient systemic infection, resulting in the impossibility to detect vaccine parasites in internal organs at long-term [34, 35]. The absence of detectable parasites in the spleen is characteristic of the infection with different attenuated viscerotropic lines and differs from the chronic infection of the spleen observed after challenge with infective parasites [36, 37], reinforcing the attenuated nature of the *LiΔHSP70-II* line. Further, a previous report indicated that the *LiΔHSP70-II* line tend to be undetectable when inoculated i.v. in immuno-deficient SCID mice [14]. These data were taken as a suggestion that parasite clearance did not strictly depends on the induction of T cell dependent responses. Something similar occurred with the *LdCen*^*-/-*^ attenuated line [35], but not with other versions of genetically modified parasites, as for example the SIR2-deficient *L*. *infantum* (*LiSIR*^*+/-*^ single knock-out for the sirTuin encoding gene) [38]. Additionally, it was reported that *LiΔHSP70-II* intra-cardiac (i.c.) inoculation of hamsters, a highly susceptible VL model by *L*. *infantum* challenge [39], generates an asymptomatic infection. The absence of clinical signs of disease was correlated to the impossibility to detect attenuated parasites in the internal organs up to 9 months after inoculation [14]. All these data may be taken as an indication of the high biosafety degree of the *LiΔHSP70-II* line. Interestingly, s.c. inoculation induced a localized infection without dissemination to internal organs, resulting in the persistence of the parasites in the DLN of the infection site in BALB/c and C57BL/6 mice. On the contrary, footpad parasite burdens decreased after infection and became undetectable at longer times. The presence of parasites in the DLN accompanied by a parasite clearance with time in the site of challenge has been also described for infective *L*. *donovani* [40] or *L*. *infantum* [41], but in these models spleen macrophages resulted chronically infected. Since mice from the s.c. and i.v. vaccinated groups showed similar long-term protection against *L*. *major* challenge, it can be hypothesized that in the i.v. vaccinated mice some parasites may persist dispersed in different internal organs, but remain undetectable perhaps due to their low number. In addition, the presence of parasites in other cell types maintaining latent infections can be also a source of parasite persistence [42, 43]. This is an important issue, since maintenance of the parasite in the vertebrate host would be assuring the maintenance of immunity in the absence of recall doses against *Leishmania* [8] or other parasitic infections [44–47]. In this regard, the persistence of parasites may be indispensable to produce a concomitant immunity maintaining the number of Teff cells [48]. Nevertheless, other cells implicated in protection, namely Tcm or tissue resident memory T (Trm) cells can persist after parasite clearance [6, 49–51]. In this context, immunization with the *LiΔHSP70-II* line elicits both Tcm cells and Teff or Tem responses. One limitation of this work is the lack of studies performed to analyze the implication of Trm cells in the observed protection, an interesting question that should be addressed in future research. On the other hand, the implication of Teff cells in the robust protection associated with vaccination was demonstrated by the data obtained in the C57BL/6 mice model. It has been described that C57BL/6 mice healed from a first infection with *L*. *major* develop concomitant immunity to re-challenge consisting in the rapid migration of IFN-γ producing Teff cells to the site of reinfection [22, 48, 52, 53]. Here, we observed that 3 days after *L*. *major* challenge, a group of IFN-γ producing CD4^+^ and CD8^+^ T cells were specifically detected in the ears and lymph node cells of vaccinated mice. These cells may correspond to pre-existing Teff cells, since Tcm cells may need more time to elicit a protective response [48]. Another limitation of our work is related with the fact that *L*. *major* was administered using a needle. However, the rapid Teff cell response demonstrated in the ear and retromandibular DLNs of C57BL/6 mice after *L*. *major* challenge may be considered as a good predictor for protection against natural challenge as occur in ‘leishmanized’ C57BL/6 mice [18].

The humoral and cellular response elicited by the BALB/c mice inoculated with the *LiΔHSP70-II* was quantitatively similar to that observed in BALB/c mice when infected i.v. or s.c. with the *L*. *infantum* infective parasites [36, 41] or to that generated after intraperitoneal inoculation with another *L*. *infantum* based attenuated line vaccine (*LiSIR2*^*+/-*^) [38]. A mixed IgG1/IgG2a humoral response, higher in titer at short-term, was observed concomitant with the systemic secretion of parasite-specific IFN-γ and IL-10 by splenocytes and a local production of SLA-dependent IFN-γ, IL-10 and IL-4 cytokines in the LN draining the site of s.c. vaccination, especially at short-term. The higher IFN-γ/IL-10 ratio showed at long-term can be correlated with clearance of the attenuated parasite, since IL-10 production has been largely related to parasite persistence of viscerotropic species [54–57]. *LiΔHSP70-II* s.c. administration to C57BL/6 mice resulted in the induction of both local and systemic Th1-like response in short- and long-term vaccinated groups, characterized by the induction of SLA-dependent IFN-γ and the presence of IgG2c anti-*Leishmania* antibodies.

The existence of susceptible and resistant models of *L*. *major* infection is an advantage when testing experimental vaccines. In the case of C57BL/6 mice, infection with a low number of metacyclic promastigotes in the dermis of the ear generates a clinically silent phase in which parasite replicates. In a second phase the IFN-γ mediated local inflammatory response reduces parasitic load leading to skin lesions similar to those of CL human patients [58]. In contrast, challenge with large numbers of parasites in the footpad of BALB/c mice generates a progressive infection associated with parasite-specific responses mediated by IL-10 and IL-4 [4, 59, 60]. Many vaccine candidates have been tested in both models with different results. In the resistant model, the protection has been linked to an anticipation of the inflammatory response, with the induction of CD4^+^ and CD8^+^ T cells producing IFN-γ which results in early control of the parasite and, therefore, in the appearance of lower grade lesions [61–64]. In the BALB/c model, numerous evidences suggest that the control of the infection not only depends on the induction of IFN-γ-mediated responses, but also on the control of IL-10 and IL-4 cytokines that are associated with pathology [29, 62, 65]. This is the case of the *LmLpg2*^*-*^ line that was able to control the pathology in BALB/c mice alleviating disease associated responses, but did not reach the same degree of protection in the resistant model when inoculated in the absence of a cellular inducing adjuvant [28, 29]. As occurred for some subunit [62] or live vaccines [31, 32], *LiΔHSP70-II* line inoculation was able to induce a robust protection in both murine models of CL. For the susceptible BALB/c mice, we first used the i.v. route, since it is the classical route of administration for viscerotropic specie based vaccines. Given that more acceptable routes of vaccination are desirable for human use the s.c. administration was also tested. Independently of the administration route and compared to unvaccinated mice, BALB/c mice inoculated with the *LiΔHSP70-II* line showed significant control of the leishmaniasis disease. This is an interesting property of our vaccine, since the Lm *dhfr-ts*^*-/-*^ line conferred protection against *L*. *major* infective challenge in BALB/c mice when it is i.v. but not s.c. administered [26]. It was also reported that protection conferred against *L*. *mexicana* by intraperitoneal inoculation of an attenuated line of the same species (lacking guanosine diphosphate-mannose pyrophosphorylase; *LmΔGDP-MP*) was not attained when it was s.c. administered [66].

Our data demonstrated that i.v. or s.c. BALB/c vaccinated mouse groups showed a Th1-like response against parasite antigens after *L*. *major* challenge. Anti-SLA humoral responses changed from the IgG1 subclass (found in the non-vaccinated controls) towards a IgG2a response. Higher IgG2a titers were observed long-term compared to short-term, correlating to a better protection degree. The parasite dependent IFN-γ response was higher in vaccinated than in control animals. The production of this cytokine was detected in short-term groups, but accompanied by the secretion of the highest levels of IL-10 among all groups. On the other hand, long-term protected mice showed a moderate SLA dependent IFN-γ production accompanied by very low parasite dependent IL-10 responses, similar to the protection conferred by the *Lmlpg2*^*-*^ parasites that was associated with control of parasite mediated IL-10 responses in this susceptible model [29].

For C57BL/6 mice, whereas the infection of non-vaccinated mice evolved as described [5, 48, 58], vaccinated mice showed no lesions at all. During the first two weeks after *L*. *major* challenge parasites similarly grew in the ear and the DLN in both vaccinated and non-vaccinated mice. Afterwards, control group continued in the silent phase incrementing their parasite burdens, while immunized mice do not allow parasites to expand further and showed earlier production of IFN-γ in the DLN. The anticipation of the effector response implies that the production of low levels of IFN-γ is sufficient to control parasite burdens without producing tissue damage. Then, *LiΔHSP70-II* parasites achieve the gold standard of protection against CL in C57BL/6 mice reaching a degree of protection comparable to that described for the more protective subunit based vaccines [61–63].

Although genetically attenuated vaccines may be an alternative to leishmanization to control human CL, concerns regarding biosafety remain, as it is mandatory that the attenuated phenotype is maintained even in cases of severe immunosuppression. In this regard, it is very important to target parasite genes whose function cannot be regained by compensatory mutations that can lead to recover the virulent phenotype to genetically modified parasites [67]. Nevertheless, the results shown in this work together with promising results observed using attenuated parasites to control malaria [68–71] and other pathologies [72–76] support the idea that live attenuated vaccines might be the basis for the development of vaccines against human CL in the next future.

## Methods

### Mice and parasites

Female BALB/c mice and C57BL/6 (6–8 weeks old) were purchased from Harlan (Barcelona, Spain). All procedures were performed according to the Directive 2010/63/UE from the European Union and RD53/2103 from the Spanish Government. Procedures were approved by the Animal Care and Use Committee at the Centro de Biología Molecular Severo Ochoa (CEEA-CBMSO 21/138), the Bioethical Committee of the CSIC (under reference 100/2014). The final approval was authorized by the Government of the Autonomous Community of Madrid under the reference PROEX121/14.

The following parasites cell lines were employed: *L*. *major* clone V1 (MHOM/IL/80/Friedlin); *L*. *infantum* (MCAN/ES/96/BCN150) and the attenuated line (*L*. *infantum* MCAN/ES/96/BCN150 [*Δhsp*70-*II*::*NEO/Δhsp70-II*::*HYG*])[12]. The attenuated line was created as described in [12]. Briefly, both alleles of the single *hsp70-II* gene located at chromosome 28 of the *L*. *infantum* genome were replaced sequentially with the ORF of the NEO and the HYG selectable marker genes by homologous recombination using plasmids constructions containing the marker genes flanked by specific regions located upstream and downstream of the ORF for *hsp70-II* gene [12]. The *LiΔHSP70-II* line showed a mild growth-rate defect in the logarithmic growth phase, concomitant with a longer duration of the G2/M phase of the cell cycle. In addition, promastigotes of the mutant line reached lower cell density than wild type parasites in culture, suffering a rapid decrease after reaching the stationary growth phase [12, 13]. Lack of functional HSP70-II gene did not affect the rate of macrophage in vitro infection but the infected macrophages showed reduced number of internal amastigotes when compared to the wild type line [13]. Parasite persistence was demonstrated in experimental infections performed in the BALB/c mice strain, since four weeks after challenge viable parasites were recovered from different organs [13, 14].

The promastigote forms of the parasites were grown at 26°C in Schneider medium (Gibco, NY, U.S.A.) supplemented with 10% Fetal Calf Serum (FCS) (Sigma, MO, U.S.A.), 100 U/ml of penicillin and 100 μg/ml of streptomycin. For the attenuated line, medium was supplemented with 20 μg/ml of G418 and 50 μg/ml of hygromycin. Parasites were kept in a virulent state by passage in BALB/c mice.

### *LiΔHSP70-II* administration, *L*. *major* challenge and parasite quantification

For vaccination, two administration routes were employed. BALB/c mice were immunized by the administration of 1 × 10^7^ *LiΔHSP70-II* promastigotes suspended in 100 μl of phosphate saline buffer (PBS) in the vein tail (intravenously; i.v.). Subcutaneously (s.c.) immunization of BALB/c and C57BL/6 mice were performed with 1 × 10^7^ *LiΔHSP70-II* promastigotes suspended in 30 μl of PBS in the right footpad. As control, mice were inoculated with PBS. In all experiments performed with BALB/c mice and in those shown in Fig 6 for C57BL/6 a single control group was employed for long- and short-term protection analyses. In these cases, mice were inoculated twice with PBS (week 12 and 4) i.v. (BALB/c) or s.c. (both mice strains). To obtain data shown in Fig 7 employing C57BL/6 mice, two different control saline groups were employed for long- or short-term analyses, receiving only one PBS dose coinciding with vaccination.

For challenge, BALB/c mice were infected with 5 × 10^4^ stationary-phase promastigotes of *L*. *major* suspended in 30 μl of PBS into the left footpads. Infection follow-up was performed by measuring footpad swelling with a metric digital caliper. Lesion size was expressed as thickness of the *L*. *major* infected left footpad minus thickness of the right footpad. C57BL/6 mice were challenged with 1 × 10^3^ (or 1 × 10^5^ when indicated) *L*. *major* metacyclic promastigotes isolated by negative selection with peanut agglutinin, suspended in 10 μl of PBS into the dermis of both ears (intradermal; i.d.). Ear lesions diameter was measured with a metric caliper.

The number of *LiΔHSP70-II* parasites was determined in the liver, spleen, BM (after i.v. or s.c. administration) and also in the DLNs and footpads after s.c. administration. In addition, *L*. *major* parasite burdens were determined in the DLNs (popliteal for BALB/c mice and retromandibular for C57BL/6 mice), ears (C57BL/6 mice) or liver and spleen (both strains). The number of parasites was determined by a limiting dilution assay as described in [77]. For cell preparation, the complete spleens, lymph nodes and footpads, or a piece of approximately 20 mg of liver were stored in Schneider medium containing 20% heat-inactivated, 100 U/ml of penicillin and 100 μg/ml of streptomycin at 4°C. Tissues were homogenized and filtered through 70 μm cell strainers (Corning Gmbh, Kaiserslautern, Germany) to obtain a cell suspension. BM samples were obtained by perfusion of the mouse femur marrow cavities with Schneider medium before filtration. For ear processing, the ventral and dorsal sheets were separated and incubated in Dulbecco's modified Eagle medium (DMEM; Thermo Fisher Scientific, MA, U.S.A.) containing Liberase CI enzyme blend (50 μg/ml; Roche Diagnostics, Basel, Switzerland). After 2 h of incubation at 37°C, the tissues were cut into small pieces, and homogenized and filtered using a cell strainer as indicated above. Each homogenized tissue sample was serially diluted (1/3) in a 96-well flat-bottomed microtiter plate containing the same medium employed for homogenization (in triplicates). For *LiΔHSP70-II* parasite number determination, medium was also supplemented by 20 μg/ml G418 and 50 μg/ml hygromycin. The number of viable parasites was determined from the highest dilution at which promastigotes could be grown up to 10 days of incubation at 26°C and is indicated per whole organ (spleen, lymph nodes and footpads), per g (liver) or as number of parasites in 10^7^ cells for the BM samples.

### Sera preparation and ELISA assays

Sera were obtained from blood samples taken before and after leishmanization with the attenuated line or after infective challenge. The reactivity against parasite proteins was determined by ELISA, using SLA prepared from *L*. *major* or *L*. *infantum* promastigotes. Briefly, SLA was prepared by three freezing and thawing cycles of stationary promastigotes suspended in PBS followed by centrifugation for 15 min at 12,000 × g using a microcentrifuge. After determining protein concentration by the Bio-Rad Protein Assay Dye Reagent (Bio-Rad laboratories, München, Germany) supernatants were collected and stored at -70°C. Sera reactivity was calculated as the reciprocal end-point titer calculated as the inverse value of the highest serum dilution factor giving an absorbance > 0.15. Briefly, MaxiSorp plates (Nunc, Roskilde, Denmark) were coated with 100 μl of SLA diluted in PBS (12 μg/ml for 12 h at 4°C). After four washes with 200 μl of PBS-Tween20 0.5% (washing buffer), wells free binding sites were blocked with the same volume of the blocking solution (PBS-Tween 20 0.5%–5% non-fat milk) for 1 h at room temperature (RT) and incubated with serial dilutions (1/2 dilution factor in blocking solution) of mouse sera for 2 h at RT. After four washes with 200 μl of washing buffer, wells were incubated for 1 h at RT with secondary antibodies. Anti-IgG, anti-IgG1, anti-IgG2a or anti-IgG2c horseradish peroxidase-conjugated anti-mouse immunoglobulins were used as secondary antibodies at 1/2,000 dilution in blocking buffer (Nordic BioSite Täby, Sweden). After four washes performed as above, the reaction was developed through incubation with orto-phenylenediamine for 10 min in the dark. Color development was stopped by the addition of 2 N H_2_SO_4_. Optical densities were read at 490 nm in an ELISA microplate spectrophotometer (Model 680, Bio-Rad Laboratories).

### In vitro cell stimulation and analysis of cytokine concentration in culture supernatants

For cytokine analysis, primary cultures were stablished from spleens and LNs as described above, but using RPMI complete medium (RPMI medium (Sigma) supplemented with 10% heat-inactivated FCS, 20 mM L-glutamine, 200 U/ml penicillin, 100 μg/ml streptomycin and 50 μg/ml gentamicin instead of Schneider medium. Cells (5 × 10^6^) were cultured during 72 h at 37°C in 5% CO_2_ in the absence or in the presence of SLA at 12 μg/ml of final concentration. The levels of IFN-γ, IL-10 or IL-4 in culture supernatants were determined by sandwich ELISA using commercial kits (Pharmingen, San Diego, CA, USA).

### Analysis of T cell populations by flow cytometry

For the analysis of effector T cells (Teff) or effector memory T cells (Tem) (CD44^+^ CD62L^low^ subset) and central memory T cells (Tcm) (CD44^+^ CD62L^high^ subset), single cell suspensions from the spleen on the BALB/c mice were processed as above, and the single splenocytes were harvested, washed in PBS with 1% heat-inactivated FCS and incubated with Rat Anti-Mouse CD16/CD32 (FcBlock, BD, Franklin Lakes, NJ, USA) followed by the staining with the surface markers: AlexaFluor 647 Rat Anti-Mouse CD3 Molecular Complex (17A2 Clone, BD), APC/Fire 750 Anti-Mouse CD44 (IM7 Clone, BioLegend, San Diego, CA, USA), BV421 anti-mouse CD62L (MEL-14 Clone, BioLegend) and BV570 anti-mouse CD4 (RM4-5 Clone, BioLegend) for 20 min at 4°C. After washing, cells were fixed and permeabilized with Cytofix/Cytoperm (BD). Finally, cells were washed and analyzed.

For identification of cell producing cytokines in BALB/c mice, single cell suspensions from the spleens or the popliteal lymph nodes of the BALB/c mice were processed as above. Subsequently, cells (1 x 10^6^) were stimulated for 2 h at 37°C in RPMI complete medium with anti-mouse CD28 (eBioscience, San Diego, CA, USA) in flat-bottom 96-well plates previously coated with anti-mouse CD3e antibody (eBioscience) 24 h before. Afterwards, 10 μg/ml Brefeldin A was added to stimulated and non-stimulated cells and incubation continued for 4 h more. Then, cells were harvested, washed in PBS with 1% heat-inactivated FCS and incubated with Fc block followed by the staining with the surface markers FITC anti-mouse CD8a (53–6.7 Clone, BioLegend), and BV570 anti-mouse CD4 for 20 min at 4°C. After washing, cells were fixed and permeabilized with Cytofix/Cytoperm (BD). Next, PE/Cy7 anti-mouse IFN-γ (XMG1.2 Clone, BioLegend) antibody was added for 30 min at 4°C. Finally, cells were washed and analyzed.

For the analysis of the frequency of T cell producing IFN-γ in the ears and retromandibular lymph nodes of C57BL/6 mice, single cell suspensions were processed 3 days after *L*. *major* challenge and 1 × 10^6^ cells were stimulated for 2 h at 37°C with anti-mouse CD3/CD28 (eBioscience) as described above. Afterwards, 10 μg/ml Brefeldin A was added and cells were incubated for 4 h more. Then, cells were washed and incubated with FcBlock followed by the staining with the surface markers FITC anti-mouse CD8a, AlexaFluor 647 Rat Anti-Mouse CD3 Molecular Complex and BV570 anti-mouse CD4 for 20 min at 4°C. After washing, cells were fixed and permeabilized with Cytofix/Cytoperm. Next, PE Rat Anti-Mouse IFN-γ (XMG1.2 Clone, BD) antibody was added for 30 min at 4°C. Finally, cells were washed and analyzed.

All cells were analyzed using a FACS Canto II flow cytometer and FACSDiva Software (BD) and processed and plotted with FlowJo Software (FlowJo LLC, Ashland, Oregon, USA).

### Statistical analysis

Statistical analysis was performed using the Graph-Pad Prism 5 program. Data were first analyzed by the D'Agostino & Pearson normality test when sample was n ≥ 8. Parametric data were analyzed by a two-tailed Student *t*-test when comparing two samples or one-way ANOVA followed by the Tukey test when comparing more than two groups. Non-parametric data (or data with n < 8) were analyzed by a Mann Whitney test or a Kruskal-Wallis test (Dunn's post-test) when comparing two or more groups, respectively. Differences were considered significant when * *P* < 0.05.

## Supporting information

S1 Fig Mice intravenously challenged with *LiΔHSP70-II* were short-term protected against *L*. *major* infective challenge. Mice (n = 8 per group) were inoculated with PBS (Saline) or with 1 × 10^7^ *LiΔHSP70-II* promastigotes in the vein tail (i.v.). At week 4 after challenge, mice (n = 8 per group) were sacrificed and the *LiΔHSP70-II* parasite burdens were determined in the spleen, liver and bone marrow. In A, scatter plots of the parasite burdens showing the mean ± standard deviation (SD) are shown. Spleen cells were cultured in the absence (Medium) or in the presence of SLA. Cytokine levels in supernatants were determined by ELISA (B). Data show the mean ± SD. * *P* < 0 .05 shows statistical differences between SLA stimulated and non-stimulated cells (unpaired Student t-test). The IgG1 and IgG2a reciprocal end-point titers against *L*. *major* SLA were analyzed at the time of the sacrifice by ELISA and represented as whisker (min to max) plots (C). * (*P* < 0.05) indicates the statistical differences between IgG1 and IgG2a anti-SLA titers (Kruskal-Wallis test and Dunn's Multiple Comparison post-test). No parasite loads or SLA-dependent antibodies or cytokines were detected in mice receiving saline. Both mice groups (n = 8 per group) were infected with 5 × 10^4^ stationary-phase *L*. *major* promastigotes in the left footpad at week 4 after vaccination. Footpad swelling was monitored weekly. Mean ± standard deviation (SD) is shown (D). *Leishmania major* parasite burdens were determined by limiting dilution in the spleen, liver and in the draining lymph node (left popliteal). Scatter plots with the individual number of parasite per total organ (spleen or lymph nodes) or per g of liver are shown including the mean ± SD (E). For D and E, * (P < 0 .05) shows the statistical differences determined by the unpaired Student t-test. Results are representative of at least two independent experiments.
(PDF)

S2 Fig Related to Fig 2. **Analysis of splenic T cell populations in vaccinated mice.** In (A) and (B) representative panels and the gating strategy and Fluorescence Minus One Control (FMO controls) of Fig 2A are shown, respectively. In (C) and (D) representative panels of Fig 2B and 2C are shown, respectively. In (E) the gating strategy and FMO controls of (C) are shown.
(PDF)

S3 Fig Determination of *LiΔHSP70-II* parasite burdens in vaccinated and *Leishmania major* BALB/c infected mice. BALB/c mice (n = 8 per group) inoculated with 1 × 10^7^ *LiΔHSP70-II* promastigotes in the vein tail (i.v.) or in the right footpad (s.c.) were infected with 5 × 10^4^ stationary-phase *L*. *major* promastigotes in the left footpad at week 4 or at week 12 after vaccination (A). Presence of the *LiΔHSP70-II* parasite burdens was determined in i.v. (B) or s.c. (C) vaccinated mice at week 20. Parasite loads were calculated by limiting dilution in the presence of G418 and hygromycin selection antibodies in the spleen, left popliteal lymph node (LP) (per total organ), in the liver (parasites per g of tissue) or in the bone marrow (BM) (parasites per 1 × 10^7^ cells) for all mice and in the right footpad (RFP) or right popliteal lymph node (RP) (per total organ) in the s.c. vaccinated mice. Scatter plots from data are shown including the mean ± standard deviation (SD).
(PDF)

S4 Fig Determination of *LiΔHSP70-II* parasite burdens in vaccinated and *Leishmania major* C57BL/6 infected mice. Presence of the *LiΔHSP70-II* parasite burdens in the spleen (Sp; parasites per total organ), liver (Liv; parasite per g), bone marrow (BM; parasites per 1 × 10^7^ cells) and right popliteal lymph node (RP; parasites per total organ) of mice immunized with the attenuated line in the right footpad before and after *L*. *major* challenge (5 weeks and 13 weeks). Parasite determinations were made at weeks 17 and 25 after vaccination in the long-term group. Parasite loads were calculated by limiting dilution in the presence of G418 and hygromycin selection antibodies. Scatter plots from data are shown including the mean ± standard deviation (SD). Results are representative of at least two independent experiments.
(PDF)

S5 Fig Related to Fig 8. **Analysis of the early response after *Leishmania major* challenge in the site of infection.** (A) and (B); gating strategy of Fig 8. (C) and (D) Fluorescence Minus One Control (FMO controls) of Fig 8.
(PDF)
