# Supplementary figures and images for: Vaccination with a Leishmania infantum HSP70-II null mutant confers long-term protective immunity against Leishmania major infection in two mice models
Source: PLoS Negl Trop Dis. 2017 May 30;11(5):e0005644. doi: 10.1371/journal.pntd.0005644 (PMC5466331; doi:10.1371/journal.pntd.0005644)

**A**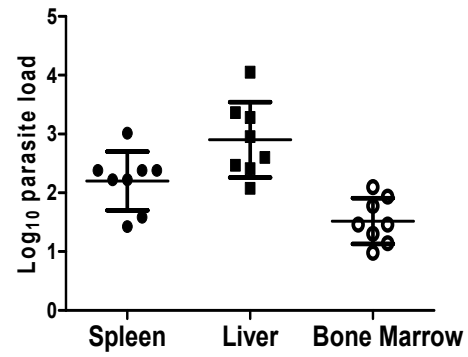**B**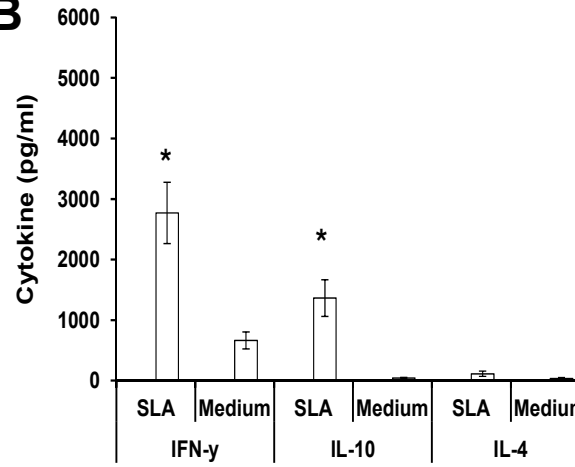**C**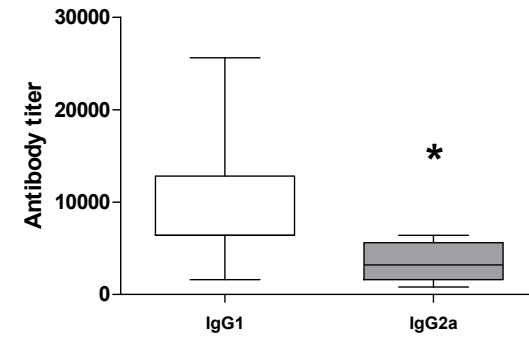**D**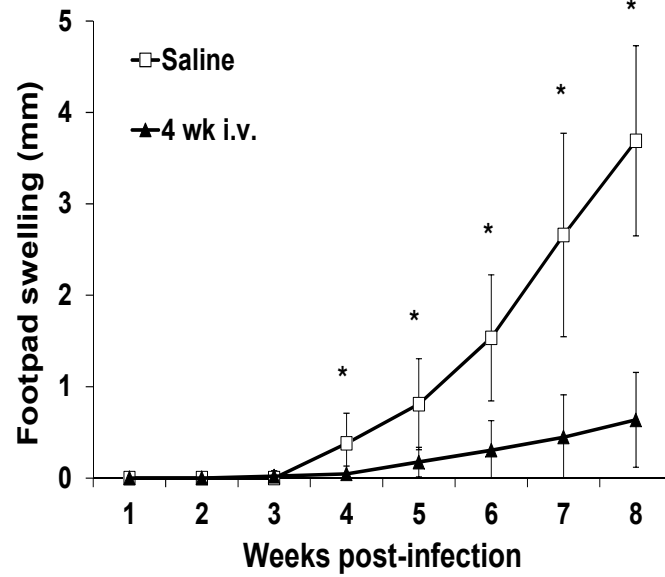**E**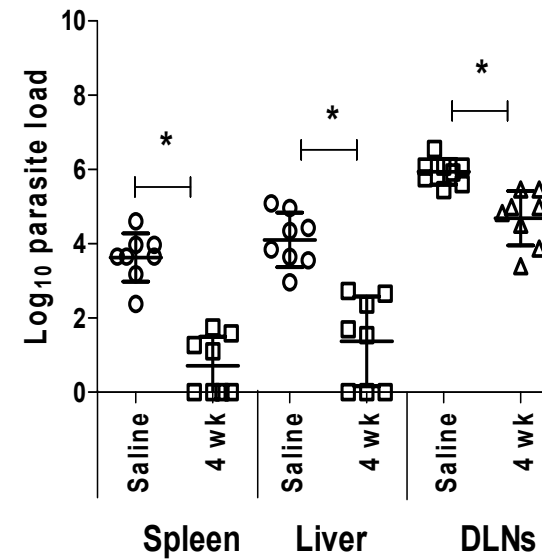

Supplement: S1 Fig — Mice (n = 8 per group) were inoculated with PBS (Saline) or with 1 × 10^7^ LiΔHSP70-II promastigotes in the vein tail (i.v.). At week 4 after challenge, mice (n = 8 per group) were sacrificed and the LiΔHSP70-II parasite burdens were determined in the spleen, liver and bone marrow. In A, scatter plots of the parasite burdens showing the mean ± standard deviation (SD) are shown. Spleen cells were cultured in the absence (Medium) or in the presence of SLA. Cytokine levels in supernatants were determined by ELISA (B). Data show the mean ± SD. * P < 0 .05 shows statistical differences between SLA stimulated and non-stimulated cells (unpaired Student t-test). The IgG1 and IgG2a reciprocal end-point titers against L. major SLA were analyzed at the time of the sacrifice by ELISA and represented as whisker (min to max) plots (C). * (P < 0.05) indicates the statistical differences between IgG1 and IgG2a anti-SLA titers (Kruskal-Wallis test and Dunn's Multiple Comparison post-test). No parasite loads or SLA-dependent antibodies or cytokines were detected in mice receiving saline. Both mice groups (n = 8 per group) were infected with 5 × 10^4^ stationary-phase L. major promastigotes in the left footpad at week 4 after vaccination. Footpad swelling was monitored weekly. Mean ± standard deviation (SD) is shown (D). Leishmania major parasite burdens were determined by limiting dilution in the spleen, liver and in the draining lymph node (left popliteal). Scatter plots with the individual number of parasite per total organ (spleen or lymph nodes) or per g of liver are shown including the mean ± SD (E). For D and E, * (P < 0 .05) shows the statistical differences determined by the unpaired Student t-test. Results are representative of at least two independent experiments. (PDF) [file pntd.0005644.s001.pdf]

**A**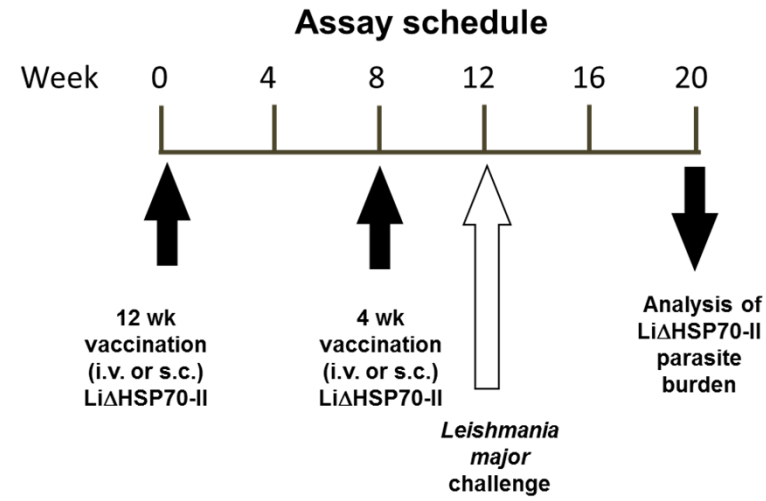**B**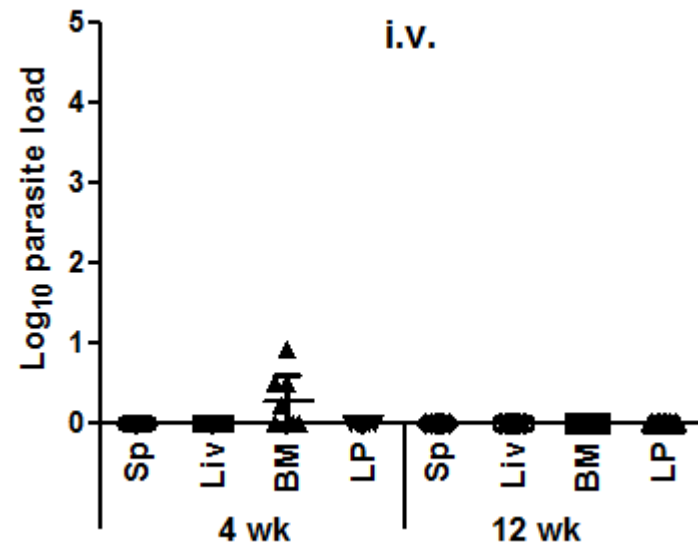**C**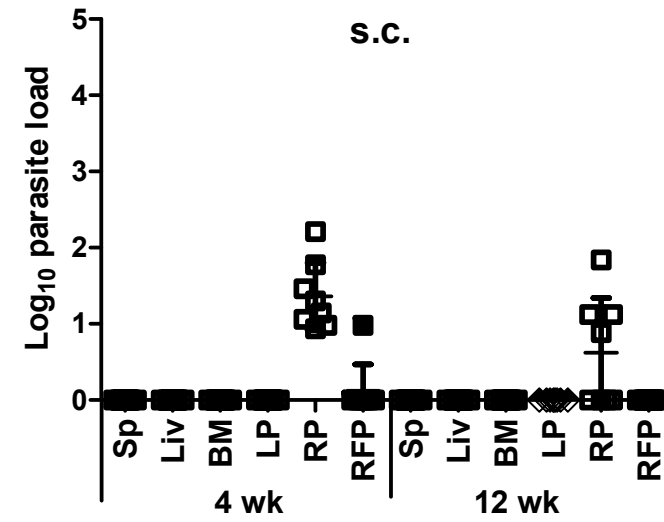

Supplement: S3 Fig — BALB/c mice (n = 8 per group) inoculated with 1 × 10^7^ LiΔHSP70-II promastigotes in the vein tail (i.v.) or in the right footpad (s.c.) were infected with 5 × 10^4^ stationary-phase L. major promastigotes in the left footpad at week 4 or at week 12 after vaccination (A). Presence of the LiΔHSP70-II parasite burdens was determined in i.v. (B) or s.c. (C) vaccinated mice at week 20. Parasite loads were calculated by limiting dilution in the presence of G418 and hygromycin selection antibodies in the spleen, left popliteal lymph node (LP) (per total organ), in the liver (parasites per g of tissue) or in the bone marrow (BM) (parasites per 1 × 10^7^ cells) for all mice and in the right footpad (RFP) or right popliteal lymph node (RP) (per total organ) in the s.c. vaccinated mice. Scatter plots from data are shown including the mean ± standard deviation (SD). (PDF) [file pntd.0005644.s003.pdf]

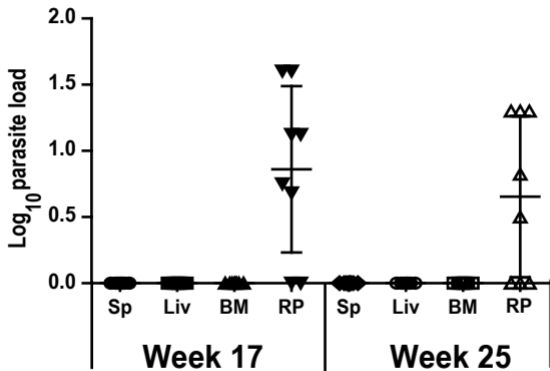

Supplement: S4 Fig — Presence of the LiΔHSP70-II parasite burdens in the spleen (Sp; parasites per total organ), liver (Liv; parasite per g), bone marrow (BM; parasites per 1 × 10^7^ cells) and right popliteal lymph node (RP; parasites per total organ) of mice immunized with the attenuated line in the right footpad before and after L. major challenge (5 weeks and 13 weeks). Parasite determinations were made at weeks 17 and 25 after vaccination in the long-term group. Parasite loads were calculated by limiting dilution in the presence of G418 and hygromycin selection antibodies. Scatter plots from data are shown including the mean ± standard deviation (SD). Results are representative of at least two independent experiments. (PDF) [file pntd.0005644.s004.pdf]
