# Supplementary material for: Vaccination with a Leishmania infantum HSP70-II null mutant confers long-term protective immunity against Leishmania major infection in two mice models
Source: PLoS Negl Trop Dis. 2017 May 30;11(5):e0005644. doi: 10.1371/journal.pntd.0005644 (PMC5466331; doi:10.1371/journal.pntd.0005644)
Supplement: S2 Fig — Analysis of splenic T cell populations in vaccinated mice. In (A) and (B) representative panels and the gating strategy and Fluorescence Minus One Control (FMO controls) of Fig 2A are shown, respectively. In (C) and (D) representative panels of Fig 2B and 2C are shown, respectively. In (E) the gating strategy and FMO controls of (C) are shown. (PDF) [file pntd.0005644.s002.pdf]

A

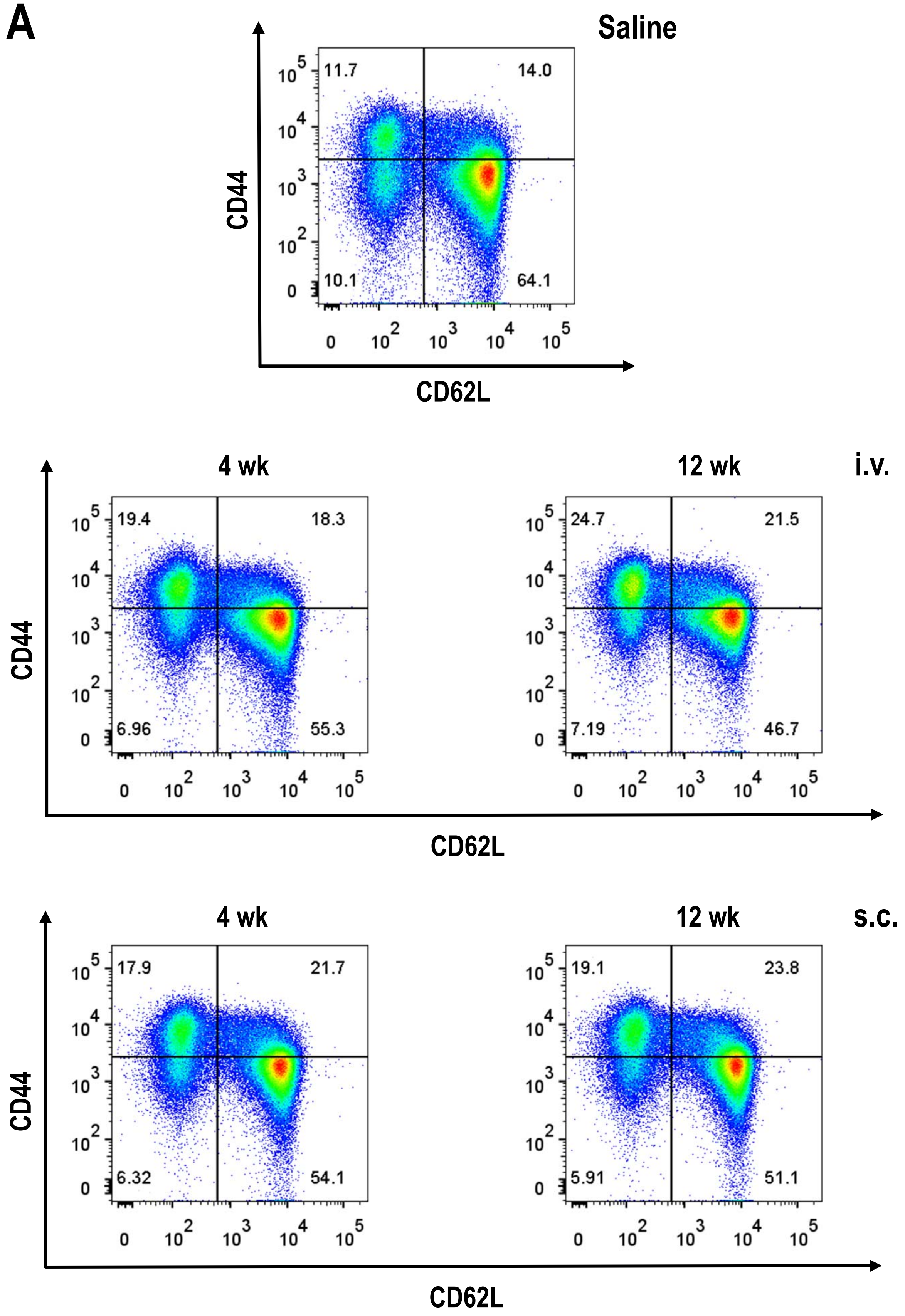

Saline
CD44
CD62L
11.7
14.0
10.1
64.1
4 wk
12 wk
i.v.
19.4
18.3
6.96
55.3
24.7
21.5
7.19
46.7
s.c.
17.9
21.7
6.32
54.1
19.1
23.8
5.91
51.1

B

Gating strategy

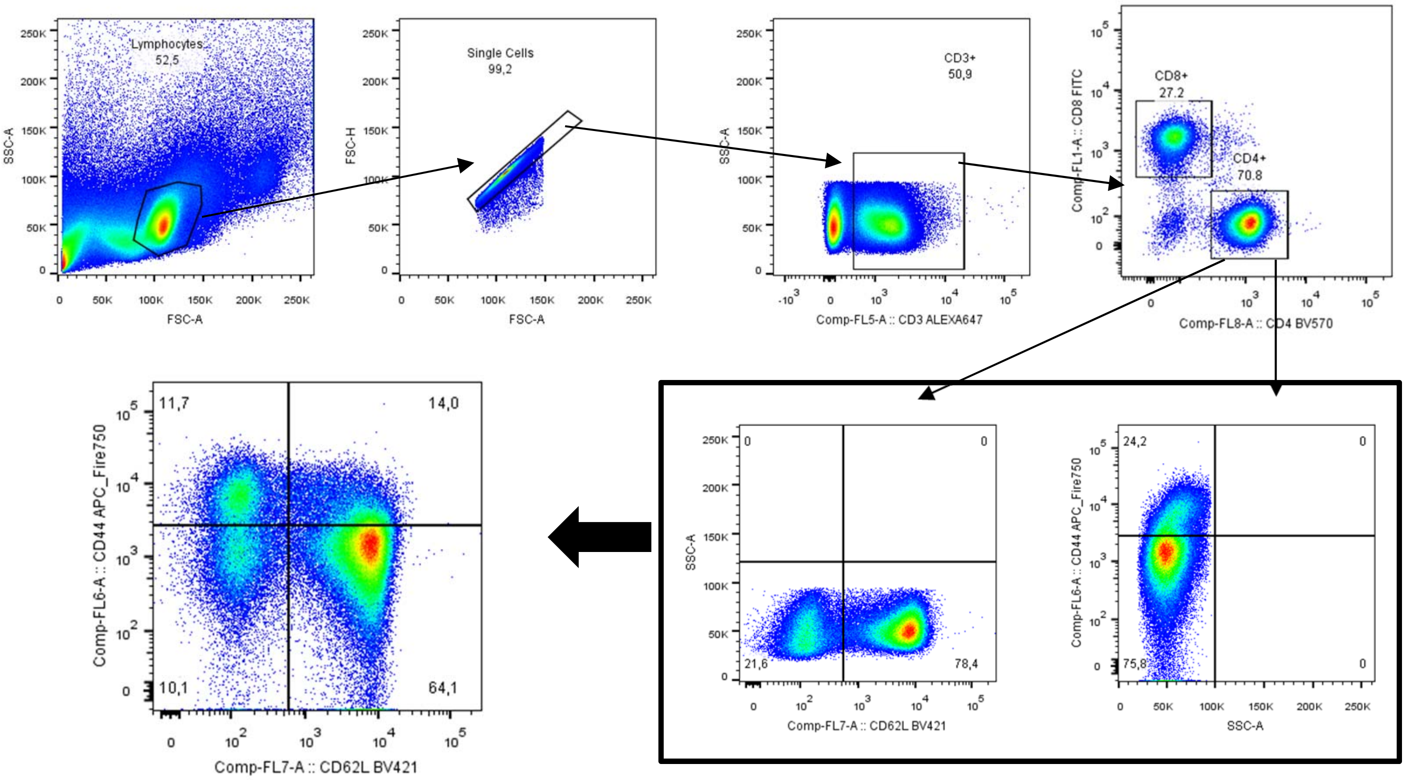

FMO Controls

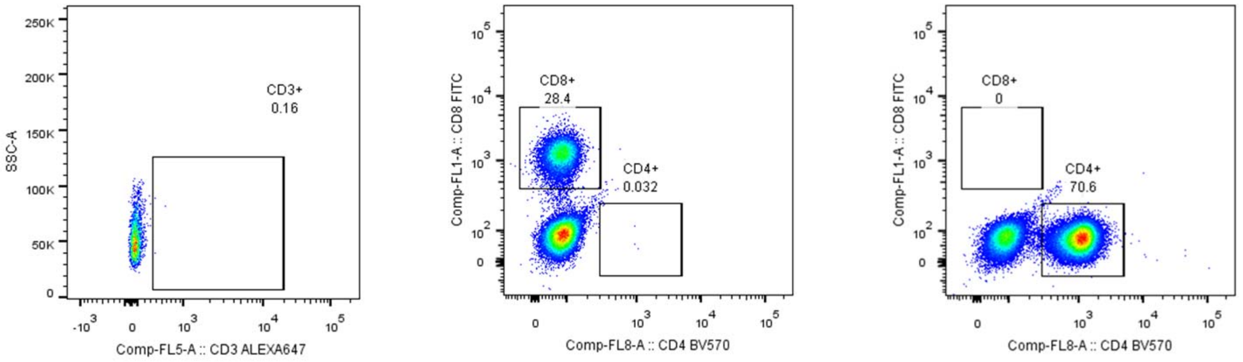

FMO CD3 **FMO CD4** **FMO CD8**

C

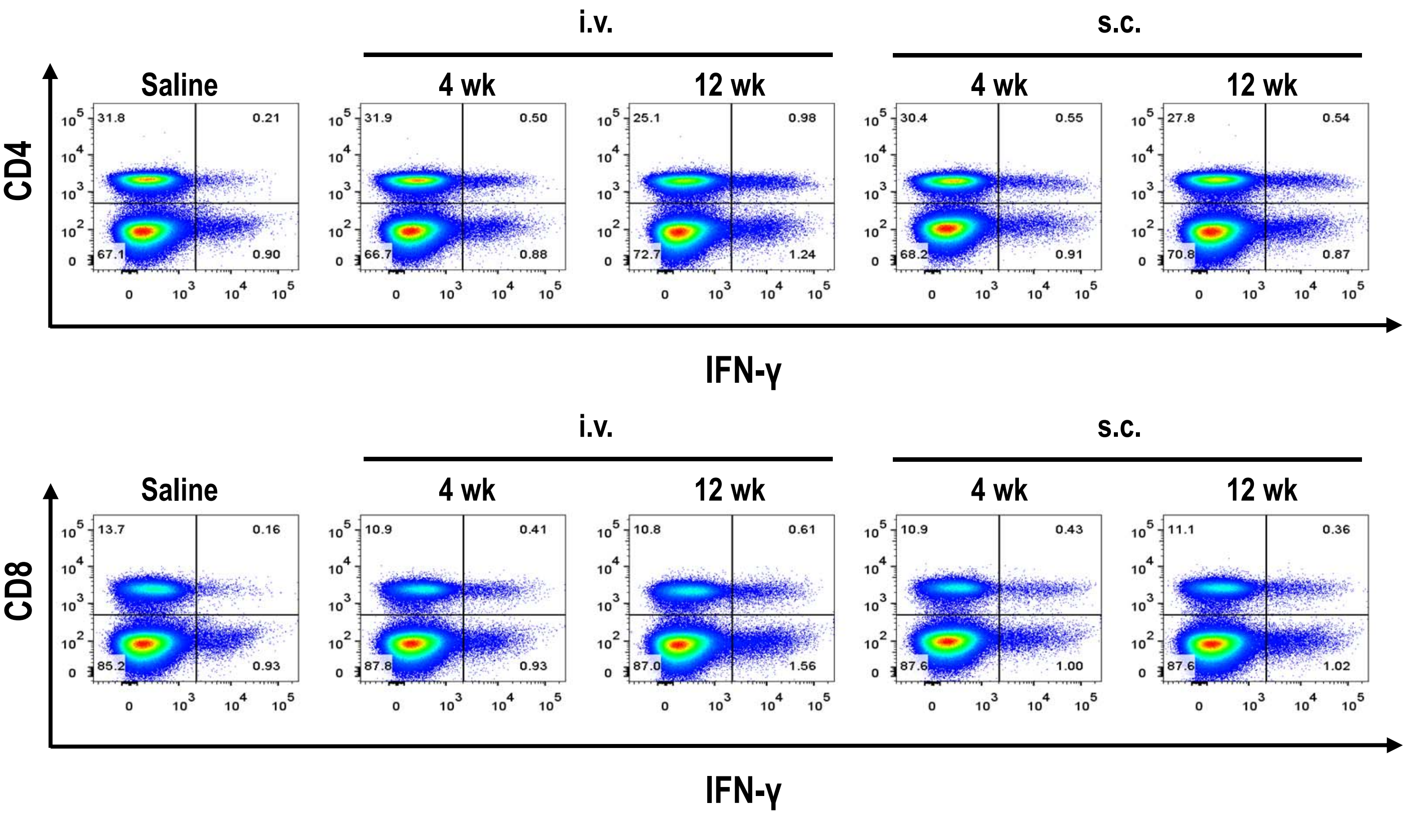

i.v.
s.c.
Saline
4 wk
12 wk
4 wk
12 wk
CD4
31.8
0.21
67.1
0.90
31.9
0.50
66.7
0.88
25.1
0.98
72.7
1.24
30.4
0.55
68.2
0.91
27.8
0.54
70.8
0.87
IFN-γ
i.v.
s.c.
Saline
4 wk
12 wk
4 wk
12 wk
CD8
13.7
0.16
85.2
0.93
10.9
0.41
87.8
0.93
10.8
0.61
87.0
1.56
10.9
0.43
87.6
1.00
11.1
0.36
87.6
1.02
IFN-γ

D

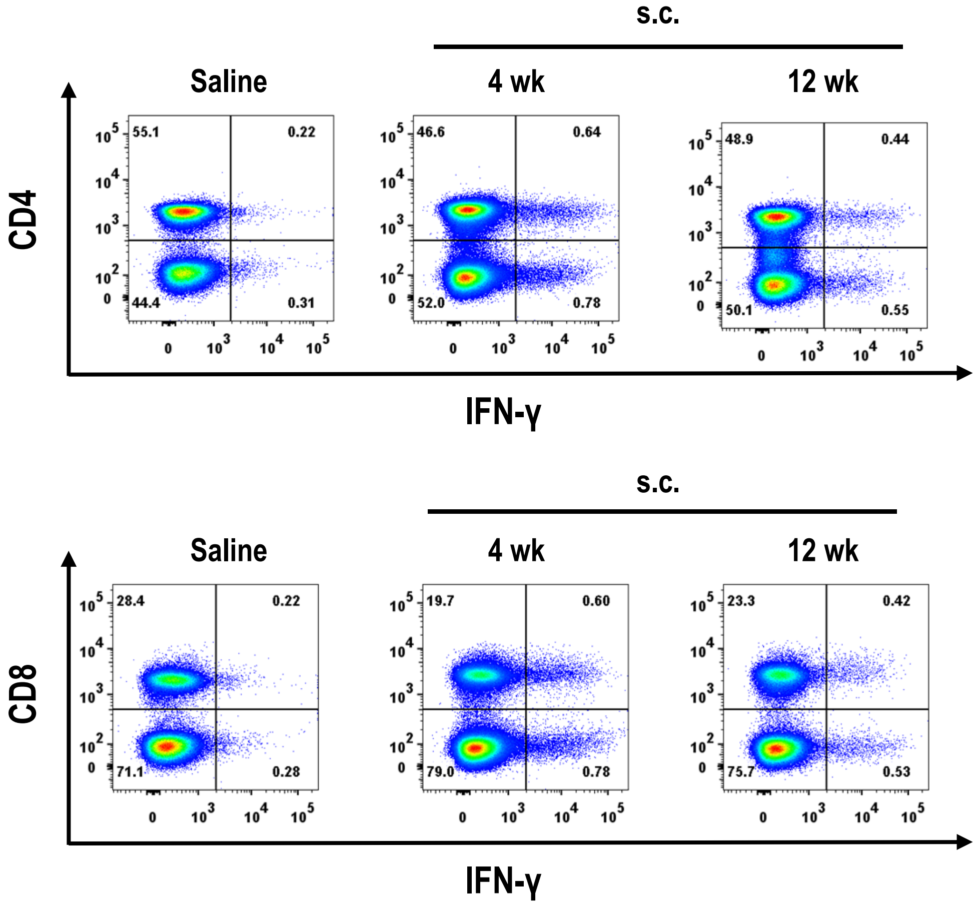

s.c.
Saline
4 wk
12 wk
CD4
55.1
0.22
44.4
0.31
46.6
0.64
52.0
0.78
48.9
0.44
50.1
0.55
IFN-γ
s.c.
Saline
4 wk
12 wk
CD8
28.4
0.22
71.1
0.28
19.7
0.60
79.0
0.78
23.3
0.42
75.7
0.53
IFN-γ

E

Gating strategy

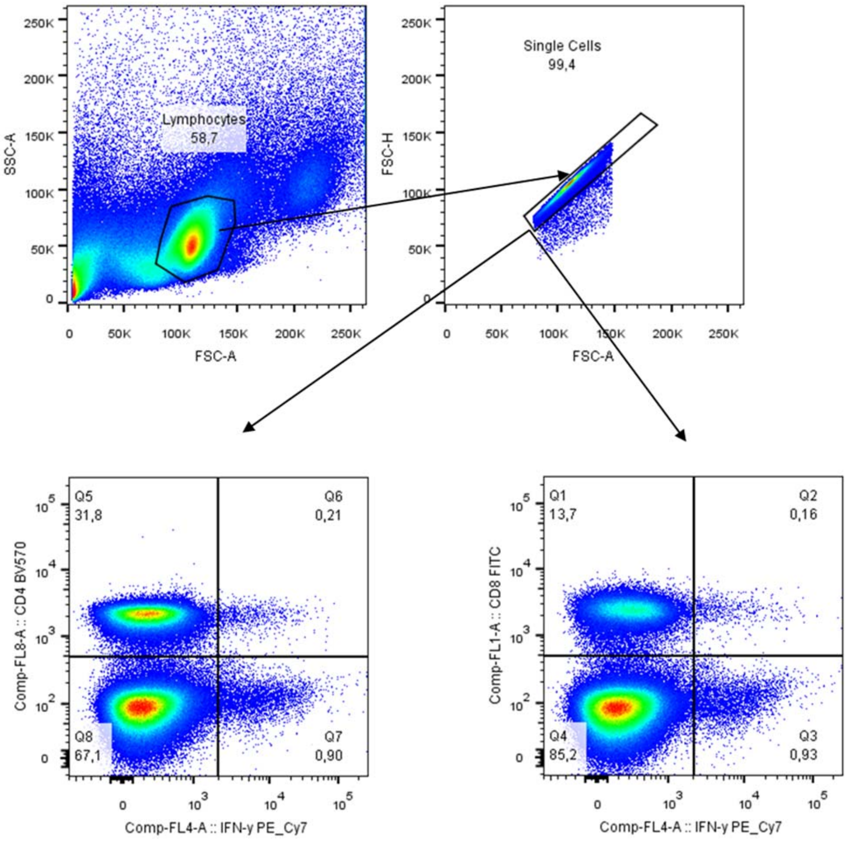

FMO Controls

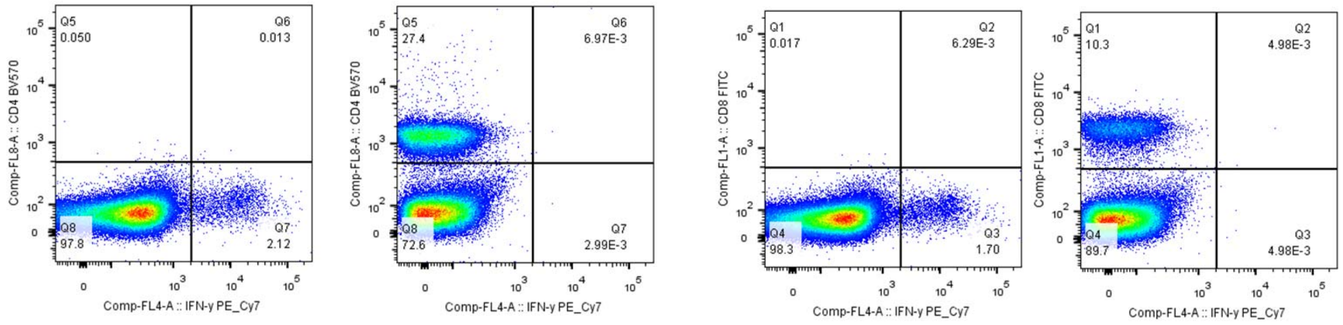

FMO CD4 **FMO IFN-γ** **FMO CD8** **FMO IFN-γ**
