# Supplementary material for: Vaccination with a Leishmania infantum HSP70-II null mutant confers long-term protective immunity against Leishmania major infection in two mice models
Source: PLoS Negl Trop Dis. 2017 May 30;11(5):e0005644. doi: 10.1371/journal.pntd.0005644 (PMC5466331; doi:10.1371/journal.pntd.0005644)
Supplement: S5 Fig — Analysis of the early response after Leishmania major challenge in the site of infection. (A) and (B); gating strategy of Fig 8. (C) and (D) Fluorescence Minus One Control (FMO controls) of Fig 8. (PDF) [file pntd.0005644.s005.pdf]

A Gating strategy; LNCs

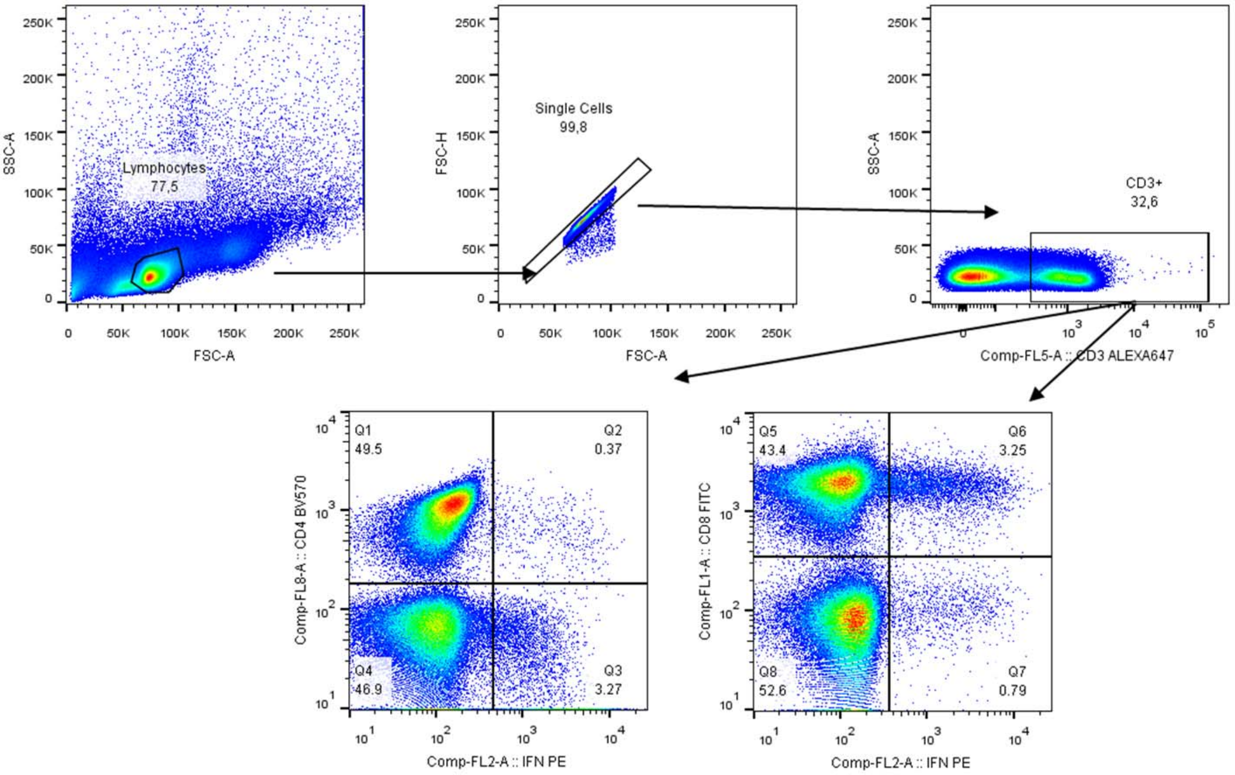

B Gating strategy; Ear

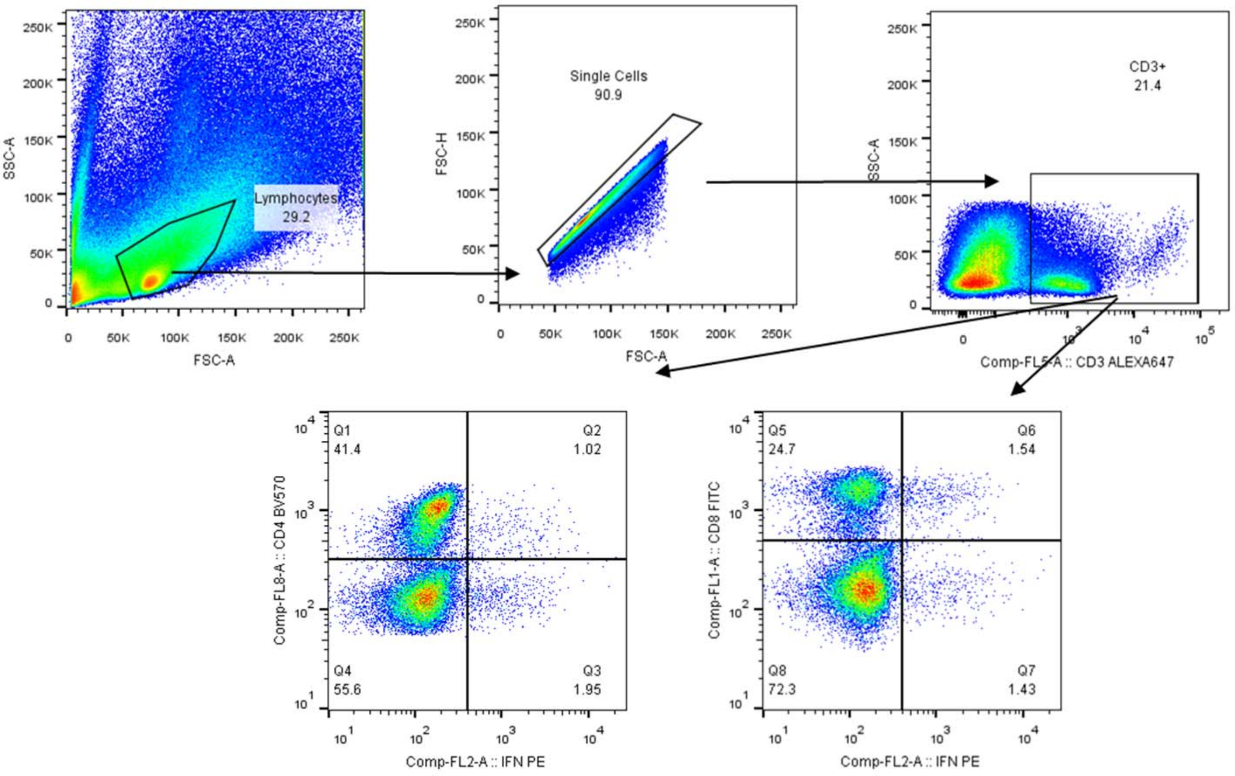

C **FMO Controls LNCs**

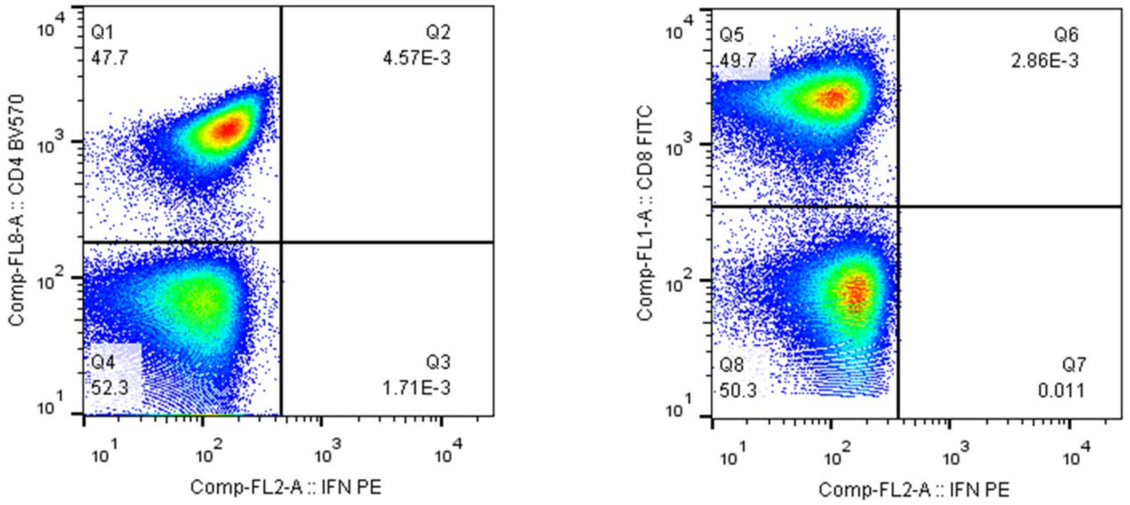

FMO IFN-γ **FMO IFN-γ**

D **FMO Controls Ear**

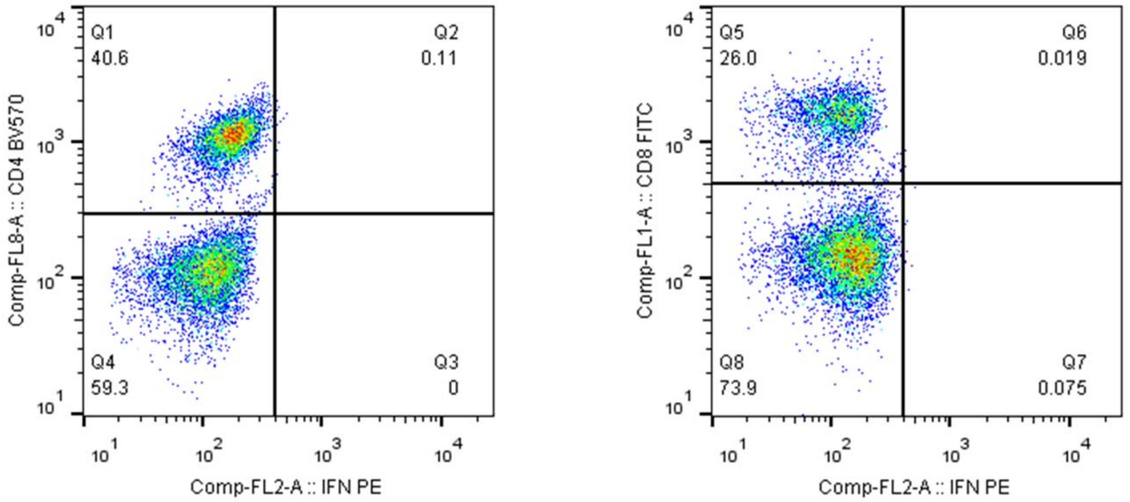

FMO IFN-γ **FMO IFN-γ**
